# Stress response in tomato as influenced by repeated waterlogging

**DOI:** 10.3389/fpls.2024.1331281

**Published:** 2024-07-23

**Authors:** Sonja Umićević, Biljana Kukavica, Ivana Maksimović, Uroš Gašić, Milica Milutinović, Marina Antić, Danijela Mišić

**Affiliations:** ^1^ Institute of Genetic Resources University of Banja Luka, Banja Luka, Bosnia and Herzegovina; ^2^ Faculty of Science and Mathematics University of Banja Luka, Banja Luka, Bosnia and Herzegovina; ^3^ Faculty of Agriculture, University of Novi Sad, Novi Sad, Serbia; ^4^ Department of Plant Physiology, Institute for Biological Research “Siniša Stanković”-National Institute of Republic of Serbia, University of Belgrade, Belgrade, Serbia; ^5^ Faculty of Agriculture, University of Banja Luka, Banja Luka, Bosnia and Herzegovina

**Keywords:** waterlogging, tomato genotypes, phenolics, fruits, leaves

## Abstract

**Introduction:**

Plants respond to water stress with a variety of physiological and biochemical changes, but their response varies among species, varieties and cultivars. Waterlogging in tomato reduces plant growth, degrade chlorophyll and increase concentration of oxidative parameters. Priming can alleviate stress in plants caused by waterlogging enabling plants to be more tolerant to an additional stress in the current or even subsequent generation. The aim of this study was to evaluate tomato genotypes for their sensitivity to waterlogging stress applied during early vegetative growth and at full flowering stage.

**Materials and methods:**

The study included two local genotypes, Trebinjski sitni (GB1126) and Žuti (GB1129), and the reference variety Novosadski jabučar (NJ), which is the variety most commonly used in Serbia and Bosnia and Herzegovina. The activity of class III peroxidase (POX), hydrogen peroxide (H_2_O_2_) content and malondialdehyde (MDA) content were measured spectrophotometrically, and for quantification of individual phenolic compounds, targeted approach was adopted, using UHPLC/DAD/(-)HESI-MS^2^ instrument (Dionex UltiMate 3000 UHPLC system with a DAD detector, configured with a triple quadrupole mass spectrometer TSQ Quantum Access Max (Thermo Fisher Scientific, Germany)).

**Results and discussion:**

Oxidative parameters (H_2_O_2_ and MDA) exhibited an increase in content in leaves of tomato plants that underwent waterlogging stress compared to control plants. Moreover, oxidative parameters showed positive correlation with proteins and phenolics content. The obtained correlations can indicate that one of the response strategies of tomato plants to waterlogging is the increased synthesis of proteins and phenolic compounds. The POX activity was not correlated with other parameters except with the polyphenols. A positive correlation was shown between POX activity and the content of phenolic compounds, indicating their independent roles in the removal of ROS. Changes in the phenolic profiles after the exposure of plants to waterlogging stress are recorded, and these changes were more severe in leaves and fruits of GB1129 and NJ genotypes than in GB1126. Thus, genotype GB1126 is the most efficient in maintaining the phenolic profiles of leaves and fruits, and therefore of the nutritive and organoleptic qualities of fruits following the exposure to waterlogging. Also, genotype GB1126 exhibited the ability to maintain the content of oxidative parameters during waterlogging at certain growth stages, implying certain waterlogging tolerance.

**Conclusion:**

Waterlogging triggered stress memory but not at all growth stages. The most pronounced stress memory was obtained in fruit samples in the phase of full fruit maturity on the 1^st^ truss. This study shed light on the defense mechanisms of tomato plants to repeated waterlogging stress from the perspectives of the changes in the composition of major phenolics, and pointed to the 5-*O*-caffeoylquinic acid and rutin as the chemical markers of the waterlogging stress tolerance in tomato. However, it remains to be determined whether this modulation has a positive or negative effect on the overall plant metabolism. Further investigations are needed to fully elucidate the benefits of waterlogging pretreatment in this context.

## 1 Introduction

Climate change is considered one of the greatest challenges facing humanity in the twenty-first century and is manifested in elevated precipitation patterns, global warming, saltwater intrusion, rising sea levels, and more frequent natural disasters (Trinh et al., 2021). As a result of climate change, crops are exposed to a variety of environmental stresses, affecting their growth, development, yield, and seed quality (Mellidou et al., 2021). It is estimated that globally, approximately 16% of arable land is affected by soil flooding (Ahsan et al., 2007) leading to approximately 60% yield reduction (for pea and white lupin for instance) (Pampana et al., 2016). Indeed, an example is a data record for the 120 years of observed rainfall that happened during the May of 2014 in the Balkan region providing a favorable condition for the flooding event, which caused an estimated economic damage of €3.5 billion for Serbia and Bosnia and Herzegovina (Stadtherr et al., 2016).

Based on FAOSTAT database for 2021, tomato (*Solanum lycopersicum* L.) is one of the most important horticultural crops with a substantially high economic value importance worldwide, cultivated in over 5 million hectares with a production of over 189 million tons (Food and Agriculture Organization, 2021). Moreover, tomato is grown in various climatic regions, and it is exposed to flooding/waterlogging stress caused by flash floods from nearby water bodies or excessive surface runoff during storms either in greenhouses or when cultivated in the field. Flooding can be classified as waterlogging when only the root zone is flooded or as submergence: partial (submerged roots and part of the aerial part) and complete (whole plant under water) (Sasidharan et al., 2017). During waterlogging, plants experience oxygen deprivation, which further influences stomatal closure, leaf epinasty, reduced growth, and premature leaf senescence (Rao and Li, 2003). Ethylene promotes the formation of adventitious roots stimulated by dying out of the primary root system (Sauter, 2013) but also causes flower and fruit abortion (Horchani et al., 2008). Waterlogging reduces photosynthetic activity (Irving et al., 2007), which leads to the production of reactive oxygen species (ROS: superoxide anion radical (O_2_^.−^), H_2_O_2_, and hydroxyl radical (OH.)) (Niu et al., 2023). At lower concentrations, ROS play an important role as signaling molecules; however, increased concentrations of ROS lead to damage to cellular macromolecules, which can result in cell death. The interaction of ROS with membrane lipids leads to lipid peroxidation, one of the end products of which is MDA. To control the level of ROS and to protect cells under stress conditions, plant tissues contain ROS scavenging enzymes (superoxide dismutase (SOD), catalase (CAT), peroxidases Class III (POX), ascorbate peroxidase (APX), and glutathione reductase (GR)) and a network of low-molecular weight antioxidants (ascorbate, glutathione, phenolic compounds, and α-tocopherol) (Yordanova et al., 2004; Yiu et al., 2011). Moreover, tomato plants are able to secure redox homeostasis and protection from oxidative damage through the synergetic regulation of antioxidant enzymes (Zhou et al., 2023). Phenolic compounds are extremely important antioxidants that have the ability to remove ROS by various mechanisms (by donating electrons or chelating redox active metals, as substrates for POX) (Rice-Evans et al., 1996; Michalak, 2006). Class III peroxidases are enzymes involved in the processes of growth and development of plant cells but also in antioxidant defense against various types of stress (Veljović Jovanović et al., 2018). According to Horchani et al. (2008), root hypoxia can modify the organoleptic and nutritional qualities of tomato fruit with the decrease in ascorbate and major amino acid contents—mainly glutamine and glutamate. Waterlogging in tomato reduced plant growth, degraded chlorophyll, and increased concentration of malondialdehyde and hydrogen peroxide that deteriorated membrane integrity, as well as increased catalase and peroxidase activities (Rasheed et al., 2018). Also, Yin et al. (2023) reported an increase in MDA and H_2_O_2_ content in leaves of tomato as a response to waterlogging stress. It is shown that the fruits from flooded plants produced ethylene earlier after harvest and responded with an accelerated ripening and thus softening of climacteric fruits (i.e., peaches) (Insausti and Gorjón, 2013). In order to reestablish the root-to-shoot ratio after the waterlogging, recovery involves the allocation of carbon to roots for preferential root growth (Cotrozzi et al., 2021). The damage in tomato plants caused by waterlogging stress can be alleviated by stress priming (Zhou et al., 2022). Priming can induce stress memory that enables plants to be more tolerant to an additional stress in the current or even subsequent generation; however, to date, the epigenetic regulatory pathways of waterlogging memory and the likely involvement of transcriptional memory in the waterlogging recovery remain unexplained (Liu et al., 2021). Except with the priming, flood tolerance may be improved through breeding efforts such as interspecific crosses or the identification of additive or over dominance genes to expand the range and use of this crop (Witt et al., 2022).

However, relatively little is known about the response of tomato plants to repeated waterlogging stress (Niu et al., 2023). Looking for plant genotypes that show tolerance to lack of oxygen is a way to contribute to the development of tolerance to waterlogging (Ezin et al., 2012), as well as to plant stress memory in repeated waterlogging treatment. Several studies provided evidence for stress memory in plants, reporting that waterlogging during the vegetative stage can efficiently improve the tolerance of plants during the reproductive stage (Li et al., 2011). Hence, the aim of this research is to evaluate the impact of waterlogging on oxidative (H_2_O_2_ and MDA) and antioxidative parameters (phenolic compounds and POX activity) of tomato genotypes to determine the degree of oxidative damage and the role of antioxidants in the response to flooding. In addition, plant stress memory in repeated flooding treatment was investigated. We used two tomato genotypes from the Gene Bank of the Republic of Srpska—*Trebinjski sitni* (GB1126) and *Žuti* (GB1129) and one reference variety *Novosadski jabučar* (NJ), which is most used in the Serbia and Bosnia and Herzegovina region for the research. Genotype GB1126 has indeterminate growth type and standard leaf type with red, high rounded fruit with round cross-sectional shape, whereas genotype GB1129 has indeterminate growth type and standard leaf type with yellow, rounded fruit with both round and irregular cross-sectional shape (Rašeta et al., 2022b).

## 2 Materials and methods

### 2.1 Plant material

**Figure 1:**
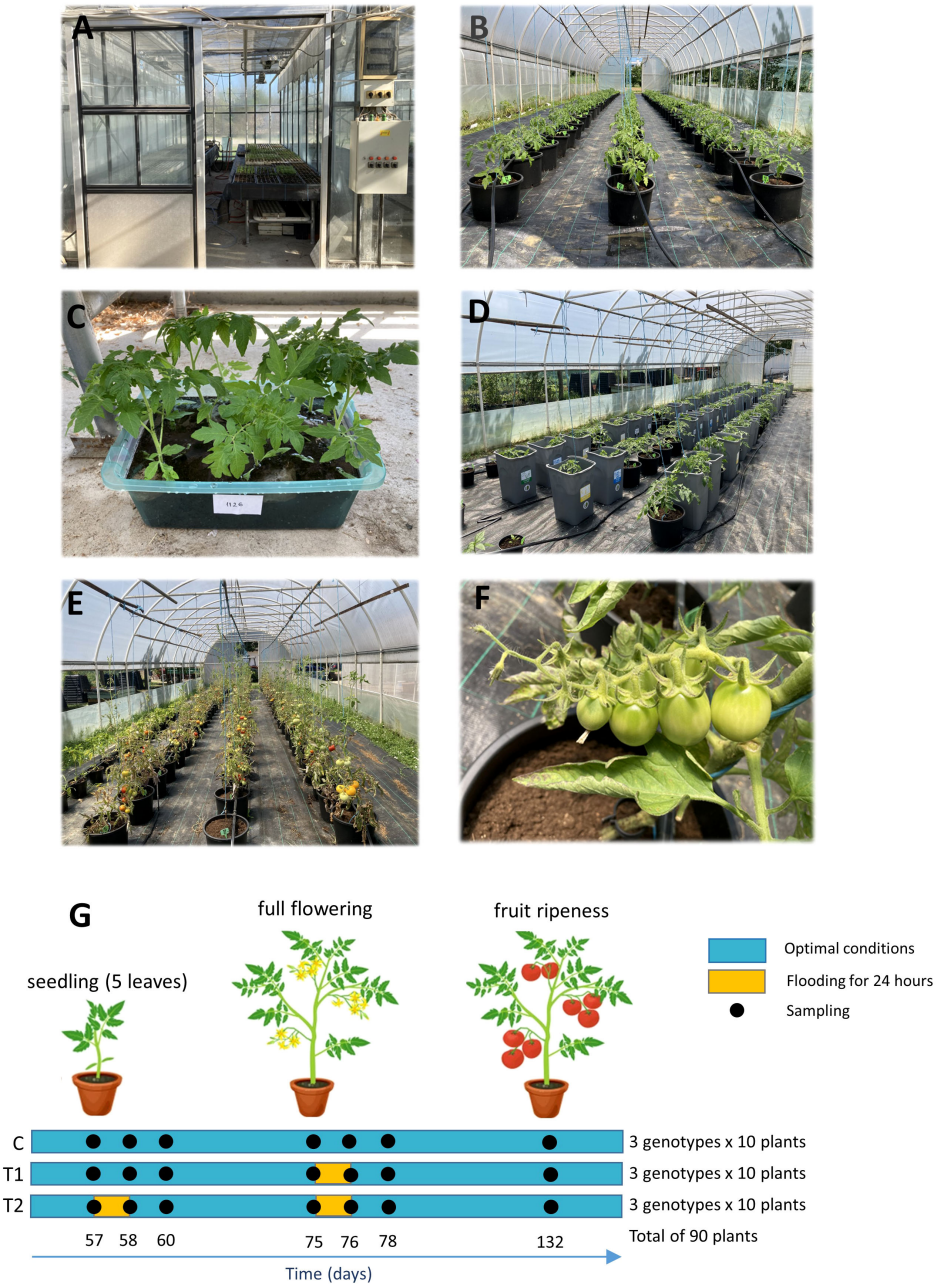
Tomato plants at the seedling stage **(A)** and during vegetation **(B)**; tomato plants under 1^st^ waterlogging treatment **(C)** and under 2^nd^ waterlogging treatment **(D)**; tomato plants at the stage of full ripeness of fruits on the 1^st^ truss **(E, F)**, scheme of the experimental setup **(G)**. C-control; T1 - one waterlogging treatment; T2 - two waterlogging treatments.

Tomato seeds were obtained from the Gene Bank of the Republic of Srpska (Banja Luka, Bosnia and Herzegovina). The present study included two local genotypes, *Trebinjski sitni* (GB1126) and *Žuti* (GB1129), and the reference variety *Novosadski jabučar* (NJ), which is the variety most commonly used in Serbia and Bosnia and Herzegovina. Containerized seedlings were produced according to standard agricultural technology in the unheated glass greenhouse at the Faculty of Agriculture, University of Banja Luka (**Figure 1A**); seeds were sown on 10/03/2022; and seedlings were pricked out on the 13th of April and uprooted to pots on the 11th of May. The experiment lasted until the 18th of July.

The experiment was established in a tunnel-type polypropylene greenhouse at the Institute of Genetic Resources, University of Banja Luka (158-m altitude, 44.774971 latitude and 17.211463 longitude), with a total area of 115 m^2^. 50 days after sprouting, seedlings were planted in polyethylene pots (28-cm diameter; 15 L) filled with a Klasmann TS3 substrate (Germany). This substrate is moderately decomposed white peat with particle size 0 mm–25 mm, pH (H_2_O, v/v 1:2:5) 6.0, water capacity 75%–80%, and air capacity 10%–15%. The substrate contains nutrients added: 140 mg/L N, 160 mg/L P_2_O_5_, 180 mg/L K_2_O, 100 mg/L Mg, and all necessary trace elements. The substrate temperature in the pots and the air temperature in the greenhouse were directly influenced by the external weather conditions logged every day on an outside meteorological station next to the greenhouse, which is depicted in **Supplementary Table 1** (The Republic Hydrometeorological Institute, 2022) and photoperiod depicted in **Supplementary Table 2** (Tutiempo Network, S. L., 2022).

A total of 90 plants were planted, with 30 plants per genotype, which were further divided into 3 growth regimes with 10 replications. Plants were randomly ordered with spacing between plants 90 × 45 cm and a drip irrigation system installed. Irrigation pressure was set at 1.5 Bar with the output of 5 L/m/h. Foliar fertilizer Fitofert Liquid 12–4-6 (Fertico, Serbia) was applied twice during vegetation (80th and 110th days of growing) in concentration 3 mL per 1 L of water using a handheld sprayer. The fertilizer contains <25% CH_4_N_2_O, <6% KNO_3_, <8% KH_2_PO_4_ with microelements (Fe, Mn, Yn, Cu, B, and Mo) and pH 6.8 and EC 0.2. Plants were maintained using standard horticultural practices such as trellising and pinching (**Figure 1B**).

### 2.2 Experimental design

Tomato plants were divided into three growth regimes: treatment 2 (T2), treatment 1 (T1), and control (C). T2 plants underwent two waterlogging treatments: pretreatment at the stage of seedlings with five fully developed leaves—BBCH-scale 105 (Meier, 2001) (**Figure 1C**) and second treatment at the stage of full flowering on the first truss—BBCH-scale 601 (Meier, 2001) (**Figure 1D**). T1 plants underwent only one waterlogging treatment, at the stage of full flowering on the first truss. For waterlogging treatment, potted plants were placed in the larger pots filled with water at level 5 cm–10 cm above the surface of soil. Both waterlogging treatments lasted for 24 h after which plants were left to drain completely. Control plants were grown under regular non-waterlogged conditions.

Sampling was done seven times during growing; prior to, 1 day after, and 3 days after the first waterlogging treatment (57th, 58th, and 60th days of growing); prior to, 1 day after, and 3 days after the second waterlogging treatment (75th, 76th, and 78th days of growing); and in the stage of full ripeness of the fruits on the first truss—BBCH-scale 809 (Meier, 2001) (132nd day of growing) (**Figures 1E–G**). The sample was made out of 10 leaves or fruits taken from 10 plants (replications). Tomato skin color was used as a marker of fruit ripening, thus ensuring that the sampled fruits were harvested at the same ripening stage. Leaf samples collected prior to, 1 day after, and 3 days after the 1st and 2nd waterlogging treatments were stored at −80°C as a fresh material. Leaf samples collected in the stage of full ripeness of fruits on the first truss were freeze dried and stored at −80°C. Fruit samples for phenolic content were freeze dried and stored at −80°C, whereas fruit samples for other parameters of antioxidative response were stored at −80°C as a fresh material.

### 2.3 Waterlogging tolerance tests

One week after the first and second waterlogging treatments, tomato plants were visually scored for tolerance (TOL) according to Yeboah et al. (2008) using a scale of 0–5, where 0 = dead plant, 1 = 100%–75% wilting, 2 = 74%–50% wilting, 3 = leaves between base and middle undulating and recurved, 4 = recurved leave margins, and 5 = green plant with no sign of stress. The higher scale stood for tolerance, whereas the lower scale stood for susceptibility. Adventitious root formation (ARF) according to modified scale by Yeboah et al. (2008) was scored visually with 0 and 1, where 0 = none, 1 = present. Moreover, yellow leaf percentage (YLP) according to Ezin et al. (2010), modified from Mohanty and Ong (2003), was visually determined using a scale of 1–6, where 1 = no yellow leaves, 2 = 10%–30% of yellow leaves, 3 = 30%–50% of yellow leaves, 4 = 50%–70% of yellow leaves, 5 = most yellow leaves, 6 = all yellow leaves.

### 2.4 Oxidative parameters (H_2_O_2_ and MDA)

The H_2_O_2_ content was determined according to Sergiev et al. (1997). Plant tissue (1 **g**) was homogenized with 7 mL 0.1% trichloroacetic acid (TCA) and centrifuged for 20 **min** at 10,000 rmp. The supernatant (0.5 mL) was mixed with 0.5 mL 10 mM NaPi (pH 6.8) and 1 mL of 1 M KI in triplicate. The absorbance was measured at 390 nm using a UV–VIS spectrophotometer (Shimadzu UV-160, Kyoto, Japan), and results were expressed in μmol g FW^−1^ for leaf samples collected prior to, 1 day after, and 3 days after the first and second waterlogging treatments and for fruit samples, whereas results are expressed as μmol g DW^−1^ for leaf samples collected in the stage of full ripeness of fruits on the first truss.

The MDA content was measured by Heath and Packer’s method (Heath and Packer, 1968). Plant tissue (1 **g**) was homogenized with 7 mL of 0.1% TCA and then centrifuged for 20 **min** at 10,000 rmp. The reaction mixture prepared in triplicate containing 1 mL of 0.5% of 2-thiobarbituric acid (TBA) in 20% TCA and 0.5 mL supernatant was heated in a hot water bath at 95°C for 30 **min**. After that, the mixture was cooled on ice and centrifuged for 10 **min** at 10,000 rmp, and the absorbance was measured at 532 nm and 600 nm using a UV–VIS spectrophotometer (Shimadzu UV-160, Kyoto, Japan). MDA content was calculated using the extinction coefficient 155 mM^−1^ cm^−1^, and results were expressed as μmol g FW^−1^ for leaf samples collected prior to, 1 day after, and 3 days after the first and second waterlogging treatments and for fruit samples, whereas results are expressed as μmol g DW^−1^ for leaf samples collected in the stage of full ripeness of fruits on the first truss.

### 2.5 Protein extraction and determination of total protein content

Tomato samples were powdered in liquid nitrogen to a fine powder. For protein extraction, 0.5 g of plant tissue was extracted with 4 mL of 0.1 mM Na-Pi pH 6.4 extraction buffer containing 1 mM phenylmethyl sulphonyl fluoride (PMSF), 0.2% Tween, and 1% polyvinylpyrrolidone (PVP). The homogenate was centrifuged for 15 min at 4°C at 10,000 rmp, and the supernatant containing soluble proteins was used to determine protein concentration and class III peroxidase activity. Total protein content was determined according to Lowry et al. (1951) using bovine serum albumin (BSA) as standard in the concentration range 0.1 mg–1 mg. The absorbance was measured in the supernatant using a UV–VIS spectrophotometer (Shimadzu UV-160, Kyoto, Japan) at 550 nm. All measurements were performed in triplicate, and results are expressed as mg g FW^−1^ for leaf samples collected prior to, 1 day after, and 3 days after the first and second waterlogging treatments and for fruit samples, whereas results are expressed as mg g DW^−1^ for leaf samples collected in the stage of full ripeness of fruits on the first truss.

### 2.6 Total class III peroxidase activity

Total class III peroxidase activity in samples was measured as absorbance increase at 430 nm with pyrogallol as a hydrogen donor. The reaction mixture consisted of 0.03 mL of 1 M pyrogallol, 0.01 mL of 1 M H_2_O_2_, 2.91 mL of 1 M Na–Pi (pH 6.4), and 0.05 mL of the extract. Absorbance was measured using a UV–VIS spectrophotometer (Shimadzu UV-160, Kyoto, Japan), and activity was calculated using the extinction coefficient for purpurgaline (A_430_ ϵ = 12 mM^−1^ cm^−1^) (Kukavica et al., 2009). Results were expressed as μmol min^−1^ g FW^−1^ protein for leaf samples collected prior to, 1 day after, and 3 days after the first and second waterlogging treatments and for fruit samples, whereas results are expressed as μmol min^−1^ g DW^−1^ protein for leaf samples collected in the stage of full ripeness of fruits on the first truss.

### 2.7 Methanol extract preparation

Tomato fruits and leaves from the three genotypes were excised, immediately frozen in liquid nitrogen, milled to a fine powder under cryogenic conditions, and stored at −80°C until analysis. Three biological replicates were analyzed, each consisting of a mixture of minimum three fruits or leaves. Fruit samples and leaf samples collected in the stage of full ripeness of the first truss were lyophilized, and dry samples were extracted in 96% methanol (0.1 g in 1 mL of solvent). Other samples were fresh leaves subjected to the same extraction procedure but were not lyophilized. The next day, samples were sonicated in an ultrasound water bath (Sonorex Bandelin Electronic, Germany) for 45 min and afterward centrifuged for 10 min at 10,000 rpm at 4°C. The supernatants were filtered using RC Syringe filters with 0.2-µm pore size and stored at 4°C. All extractions were performed in triplicate.

### 2.8 UHPLC/DAD/(±)HESI-MS^2^ targeted analysis

For the quantification of individual phenolic compounds, the targeted approach was adopted, using a UHPLC/DAD/(-)HESI-MS^2^ instrument (Dionex UltiMate 3000 UHPLC system with a DAD detector, configured with a triple quadrupole mass spectrometer TSQ Quantum Access Max (Thermo Fisher Scientific, Germany)). A Syncronis C18 analytical column (100 × 2.1 mm) with 1.7 µm particle size (Thermo Fisher Scientific, Germany) was used for the chromatographic separation. The mobile phase consisted of (A) water + 0.1% formic acid and (B) acetonitrile + 0.1% formic acid. A linear gradient program at a flow rate of 0.3 mL min^−1^ was used: 0.0 min–1.0 min 5% B, 1.0 min–14.0 min from 5% to 95% B, 14.0 min–14.2 min from 95% to 5% B and 5% B for 6 min. The injection volume was 5 µL. The selected reaction monitoring (SRM) mode of the instrument was used for the quantification of the targeted compounds by direct comparison with the commercial standards. All commercial standards were of analytical purity (≥95%) and purchased from Sigma-Aldrich (Darmstadt, Germany). Their average retention times, concentration ranges, molecular ions, and major MS^2^ fragments with specified collision energies, *R* ^2^, and limits of detection (LOD) and quantification (LOQ) can be found in **Table 1**. Calibration curves revealed good linearity, with *r* ^2^ values exceeding 0.99 (peak areas vs. concentration). The total amount of each phenolic compound was evaluated by calculation of the peak area and is expressed as µg per g of plant fresh (FW) for leaf samples taken prior to, 1 day after, and 3 days after the first and second waterlogging treatments or dry weight (DW) for leaf and fruit samples taken in the stage of full ripeness of fruits on the first truss.

**Table 1:** Metabolites identified in leaf and fruit samples (compound name, retention time, concentration range, mass spectral data, determination coefficient (*R* ^2^), as well as LOD and LOQ).

No	Compound name	*t*_R_, min	Concentration range, mg/mL	Molecular ion [M-H]^−^, *m/z*	Major MS^2^ fragments [M-H]^−^ (*m/z*) with specified collision energies (eV)	*R* ^2^	LOD, mg/mL	LOQ, mg/mL
**1**	3-*O*-Caffeoylquinic acid	5.00	0.01–1.00	353.103	191.28 (25)	0.9961	0.07	0.24
**2**	5-*O*-Caffeoylquinic acid	5.67	0.01–1.00	353.099	191.23 (23)	0.9951	0.08	0.27
**3**	Caffeic acid	6.18	0.05–1.00	179.079	107.34 (22); 135.14 (16)	0.9945	0.08	0.28
**4**	Rutin	6.71	0.01–1.00	609.197	299.98 (42); 301.20 (32)	0.9993	0.03	0.10
**5**	Quercetin 3-*O*-glucoside	6.96	0.01–0.75	463.002	300.02 (29); 301.04 (23)	0.9995	0.02	0.07
**6**	Naringin	7.23	0.01–1.00	579.241	271.16 (43), 459.22 (24)	0.9990	0.04	0.12
**7**	Kaempferol 3-*O*-glucoside	7.40	0.01–1.00	447.008	225.03 (43); 284.03 (29)	0.9973	0.06	0.20
**8**	Eriodictyol	8.62	0.01–0.75	286.974	135.02 (22); 150.93 (19)	0.9953	0.06	0.20
**9**	Luteolin	8.70	0.01–1.00	285.035	133.06 (36); 175.04 (27)	0.9943	0.09	0.29
**10**	Naringenin	9.39	0.01–1.00	271.077	119.10 (25); 151.07 (19)	0.9992	0.03	0.11
**11**	Apigenin	9.40	0.05–1.00	269.032	151.00 (26); 225.09 (23)	0.9955	0.08	0.25

### 2.9 Statistical analyses

To differentiate between samples, principal component analysis (PCA) was performed using the Past 4 software (version 4.12; Hammer and Harper, 2001). Moreover, hierarchical cluster analysis (HCA) plots were constructed in Morpheus software (Broad Institute, 2023), based on the Pearson or the Spearman method of cluster agglomeration, adopting the average linkage method. For the hierarchical cluster analysis (HCA), the input variables were scaled to the [0, 1] range. Quantitative data, protein content, class III peroxidase activity, and MDA and hydrogen peroxide content were subjected to *post-hoc* Tukey’s test (*P* < 0.05) of one-way ANOVA, or to Student’s *t-*tests (*P* < 0.05). Furthermore, Pearson’s correlation coefficients were used to detect the relationship between analyzed parameters.

## 3 Results and discussion

### 3.1 Tolerance to waterlogging

In most plants, including tomato, leaf yellowing and wilting are the major physiological indicators of waterlogging stress injuries (Crawford, 1982; Bhatt et al., 2015). Leaf yellowing could be due to decrease in fixation of biological nitrogen and the production of toxic substances, such as nitrites and sulfides (Kumar et al., 2013). On the other hand, ARF is considered as an important indicator of waterlogging stress tolerance/adaptation to the adverse waterlogging conditions, especially in tomato, which showed the most vigorous adventitious root growth compared with cucumber, zucchini, and bean (Walter et al., 2004).

**Figure 2:**
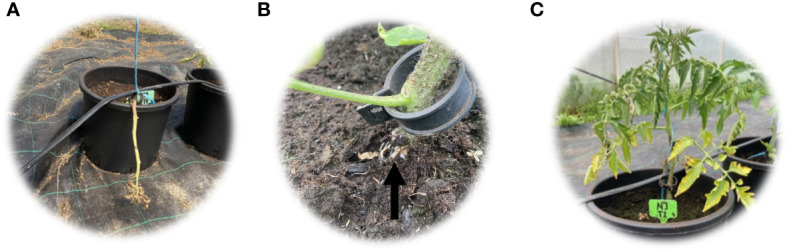
Waterlogging tolerance tests: dead plant **(A)**; adventitious root formation **(B)** and yellow leaves **(C)**.

In this work, tomato accessions were grown in greenhouse under three waterlogging regimes: control (C), one-time waterlogging (T1), and repeated waterlogging stress (T2). Seven days after the first waterlogging treatment, all plants visually appeared to be green and healthy with no sign of stress and had 10%–30% of yellow leaves, with no statistical difference between genotypes (**Table 2**, **Figure 2**). However, ARF was the most pronounced in genotype NJ (80% of treated plants), followed by GB1126 (70% of treated plants) and GB1129 (60% of treated plants) (**Table 2**). Similar to our results, Ezin et al. (2010) also reported that ARF was dependent on tomato genotype.

**Table 2:** Parameters of waterlogging tolerance tests were analyzed on all plants: 7 days after first waterlogging and 7 days after second waterlogging - tolerance (TOL) where 0 = dead plant, 1 = 100%–75% wilting, 2 = 74%–50% wilting, 3 = leaves between base and middle undulating and recurved, 4 = recurved leave margins, and 5 = green plant with no sign of stress, adventitious root formation (ARF) where 0 = absent, 1 = present, yellow leaf percentage (YLP) where 1 = no yellow leaves, 2 = 10%–30% of yellow leaves, 3 = 30%–50% of yellow leaves, 4 = 50%–70% of yellow leaves, 5 = most yellow leaves, 6 = all yellow leaves.

	7 days after first waterlogging			7 days after second waterlogging		
	TOL	ARF	YLP	TOL	ARF	YLP
GB1126—C	5.00 ± 0.00	0.00	1.00 ± 0.00	4.90 ± 0.11	0.00	1.00 ± 0.00
GB1126—T1	5.00 ± 0.00	0.00	1.00 ± 0.00	4.90 ± 0.11	1.00 ± 0.22**	1.10 ± 0.11
GB1126—T2	5.00 ± 0.00	0.70 ± 0.16**	1.00 ± 0.00	5.00 ± 0.00	1.00 ± 0.21**	1.30 ± 0.22
GB1129—C	5.00 ± 0.00	0.00	1.00 ± 0.00	4.80 ± 0.14	0.00	1.10 ± 0.11
GB1129—T1	5.00 ± 0.00	0.00	1.00 ± 0.00	4.00 ± 0.54	0.80 ± 0.21**	1.40 ± 0.17
GB1129—T2	5.00 ± 0.00	0.60 ± 0.17**	1.00 ± 0.00	4.00 ± 0.54	1.00 ± 0.22**	1.50 ± 0.36
NJ—C	5.00 ± 0.00	0.00	1.00 ± 0.00	5.00 ± 0.00	0.00	1.00 ± 0.00
NJ—T1	5.00 ± 0.00	0.00	1.00 ± 0.00	4.90 ± 0.11	0.80 ± 0.21**	1.00 ± 0.00
NJ—T2	5.00 ± 0.00	0.80 ± 0.14**	1.00 ± 0.00	4.90 ± 0.11	0.70 ± 0.22**	1.10 ± 0.11

Data represent mean ± SEM. Asterisks indicate a statistically significant difference between control and treated sample (P < 0.05, Student’s t-test).

C, control; T1, one waterlogging treatment; T2, two waterlogging treatments.

Seven days after the second waterlogging treatment, wilting was the most pronounced in genotype GB1129, as waterlogging was lethal for some individuals (**Table 2**). Genotypes GB1126 and GB1129 displayed YLP in both T1 and T2, whereas treated NJ plants did not differ from corresponding control plants (**Table 2**, **Figure 2**). These data correspond to chlorophyll loss as one of the most evident events occurring under waterlogging conditions in many flooding-sensitive species reported in the literature (Voesenek et al., 2006; Pan et al., 2021). Repeated waterlogging treatments induced ARF in GB1126 and GB1129, with genotype GB1126 having more pronounced adventitious roots (**Table 2**, **Figure 2**). Ezin et al. (2010) suggested that high ARF has the function to improve the plant’s ability to withstand the negative effects of waterlogging through obtaining oxygen directly from the air. Regardless of the genotype, in both T1 and T2 groups, 6.67% of the plants had 100%–75% wilting and 16.67% of the plants had yellow leaves to some extent. In the T1 group, 73.33% of the plants formed adventitious roots, whereas in the T2 group 80% of the plants formed adventitious roots. In the control group, wilting and ARF were not observed, whereas 3.33% of the plants had YLP. Based on these results, our study highlights that genotype GB1126 is more tolerant to waterlogging than GB1129 and NJ. Overall, different genotypes showed differing physiological responses to stress treatment.

### 3.2 Oxidative parameters (H_2_O_2_ and MDA)

**Figure 3:**
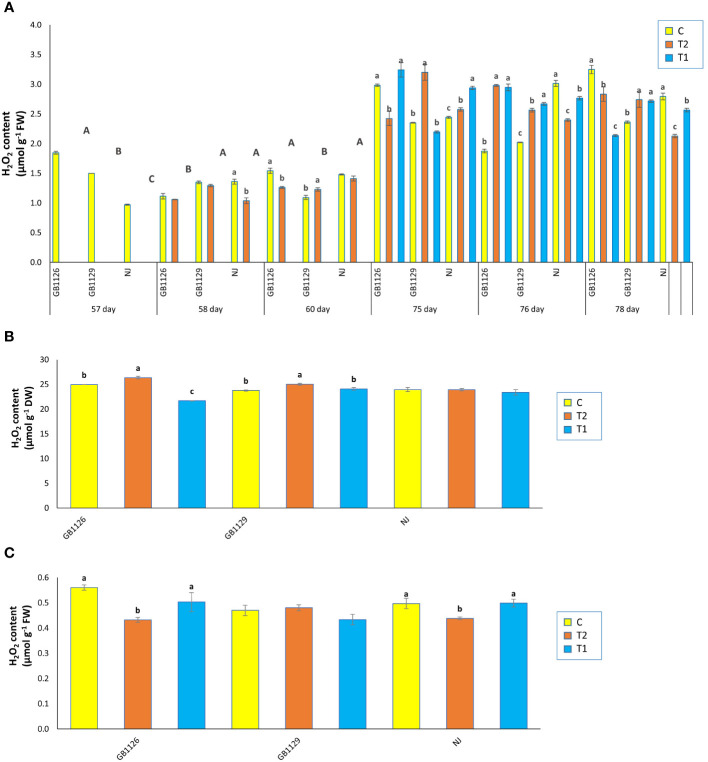
The content of hydrogen peroxide (H_2_O_2_) in leaves prior to, 1 day after and 3 days after the 1^st^ and 2^nd^ waterlogging **(A)**, in leaves at the stage of full ripeness of the fruits on the 1^st^ truss **(B)** and in fruits **(C)** of tomato genotypes GB1126, GB1129 and NJ. Data represent mean ± SEM and was analyzed by ANOVA (between treatments (a, b and c) and between genotypes (A, B and C), where different letters indicate statistical significance) followed by post hoc Tukey's test. C - control; T1 - one waterlogging treatment; T2-two waterlogging treatments.

Oxidative parameters (H_2_O_2_ and MDA) are considered to be markers of oxidative damage and exhibit plant tolerance to stress (Ashraf et al., 2012). Oxidative parameters tend to have an increased content that corresponded to increased waterlogging stress duration (Anee et al., 2019). In our research, there were significant differences in H_2_O_2_ content in leaves prior to first waterlogging among three genotypes (**Figure 3A**). Genotype GB1126 had significantly lower content of H_2_O_2_ in leaves 1 day after the first waterlogging compared with other genotypes, whereas genotype GB1129 had significantly lower content of H_2_O_2_ in leaves 3 days after the first waterlogging compared with other genotypes. Also, in genotype GB1129, the content of H_2_O_2_ significantly increased in plants that underwent waterlogging compared with control plants, which is also observed in research by Yin et al. (2023). Moreover, 1 day after the second waterlogging, both genotypes GB1126 and GB1129 showed an increase in H_2_O_2_ content in T1 and T2 compared with C, whereas genotype NJ showed a decrease in H_2_O_2_ content. However, 3 days after the second waterlogging, genotype GB1129 showed an increase in H_2_O_2_ content in T1 and T2 compared with C, whereas both genotypes GB1126 and NJ showed decrease in H_2_O_2_ content (**Figure 3A**).

In the stage of full ripeness of fruits on the first truss, genotype GB1126 showed a decrease in H_2_O_2_ content in T1 and increase in T2 compared with C, whereas genotype GB1129 showed an increase in H_2_O_2_ content only in T2 (**Figure 3B**). In fruit samples, both genotypes GB1126 and NJ showed a decrease in H_2_O_2_ content in T2 compared with T1, which could be argued that waterlogging priming induced waterlogging memory associated with H_2_O_2_ in tomato when stress reoccurred according to Niu et al. (2023) (**Figure 3C**). Also, the same authors argue that waterlogging priming induced waterlogging memory by upregulating the H_2_O_2_ content, and a high H_2_O_2_ was maintained when waterlogging reoccurred resulting in enhanced maintenance of acquired waterlogging tolerance, especially in waterlogging-sensitive tomato genotype.

**Figure 4:**
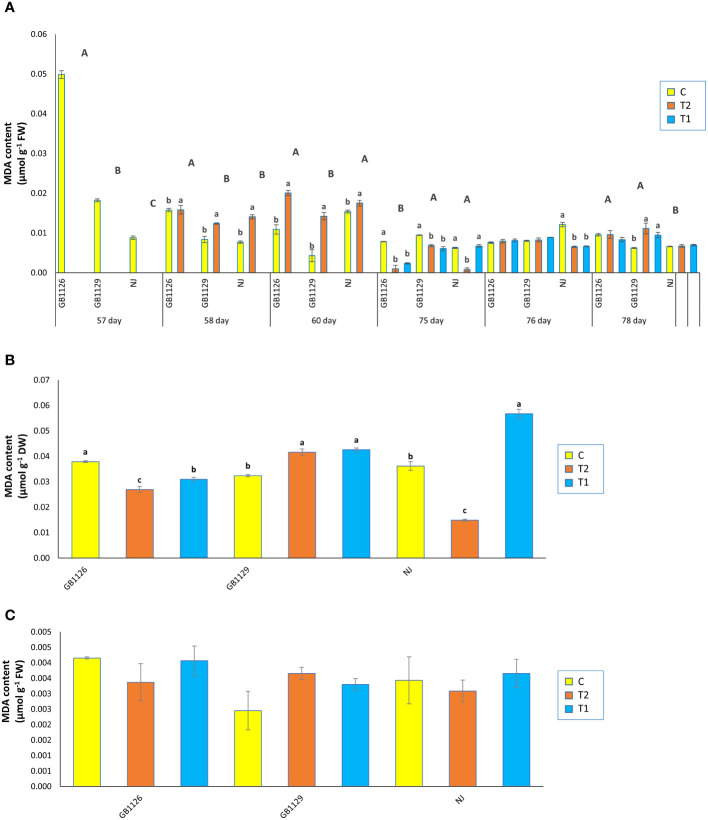
The content of malondialdehyde (MDA) in leaves prior to, 1 day after and 3 days after the 1^st^ and 2^nd^ waterlogging **(A)**, in leaves at the stage of full ripeness of the fruits on the 1^st^ truss **(B)** and in fruits **(C)** of tomato genotypes GB1126, GB1129 and NJ. Data represent mean ± SEM and was analyzed by ANOVA (between treatments (a, b and c) and between genotypes (A, B and C), where different letters indicate statistical significance) followed by post hoc Tukey's test. C - control; T1 - one waterlogging treatment; T2 - two waterlogging treatments.

MDA is the principal and most extensively studied decomposition product of membrane lipid peroxidation (Zhang et al., 2007). In our research, there was a significant difference in MDA content in leaves prior to first waterlogging among three genotypes (**Figure 4A**). Park et al. (2020) also reported differences in MDA content between genotypes. Furthermore, 1 day and 3 days after the first waterlogging treatment, all genotypes that underwent waterlogging treatment displayed increased MDA in leaves compared with control. Increased MDA content in tomato plants under waterlogging was also reported by Yin et al. (2023). Prior to second waterlogging, there was significant difference in MDA content between GB1126 and GB1129, whereas GB1129 and NJ did not differ significantly. Moreover, there was no significant difference in MDA content 1 day and 3 days after the second waterlogging treatment, except between T1 and C and T2 and C in NJ genotype 1 day after, as well as between T1 and C and T2 and C in GB1129 3 days after the second waterlogging treatment (**Figure 4A**).

In the stage of full ripeness of fruits on the first truss, genotype GB1126 showed a decrease in MDA content in T1 and T2 compared with C (**Figure 4B**). On the contrary, at the same stage, genotype GB1129 showed increase in MDA content in T1 and T2 compared with C, whereas genotype NJ showed a decrease in T2 and an increase in T1 compared with C. In fruit samples, there were no significant differences between treatments and controls in MDA content (**Figure 4C**). Waterlogging in the leaves of tomato plants leads to an increase in content of both H_2_O_2_ and MDA (Rasheed et al., 2018; Yin et al., 2023). Differences in H_2_O_2_ and MDA content between genotypes in waterlogging conditions have been discussed in the research by Niu et al. (2023).

### 3.3 The concentration of proteins

**Figure 5:**
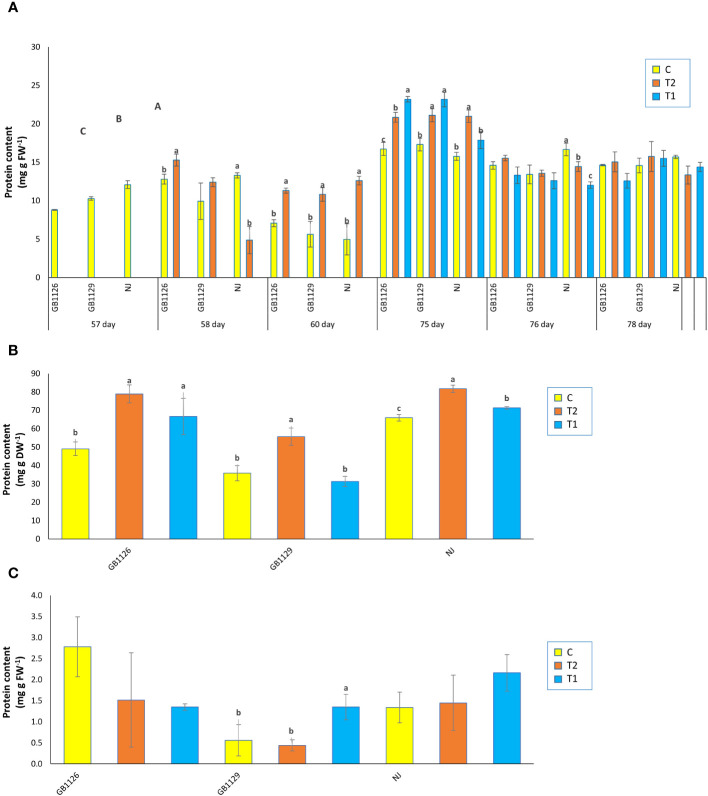
The content of proteins in leaves prior to, 1 day after and 3 days after the 1^st^ and 2^nd^ waterlogging **(A)**, in leaves at the stage of full ripeness of the fruits on the 1^st^ truss **(B)** and in fruits **(C)** of tomato genotypes GB1126, GB1129 and NJ. Data represent mean ± SEM and was analyzed by ANOVA (between treatments (a, b and c) and between genotypes (A, B and C), where different letters indicate statistical significance) followed by post hoc Tukey's test. C-control; T1 - one waterlogging treatment; T2 - two waterlogging treatments.

Stress proteins are synthesized under stress conditions as affected by stimulation of antioxidant enzymes, and its accumulation in the plant cell can keep the cytoplasmatic fluidity and osmotic potential, thereby enhancing the ability to tolerate flooding stress (Seymen, 2021). In the research by Ahsan et al. (2007), a total 52 differentially expressed proteins in leaves of tomato at the seedling stage in response to waterlogging stress, with proteins changing their intensities more than 1.5-fold. Our results showed a significant difference between three genotypes in protein content of leaves prior to the first waterlogging treatment (**Figure 5A**). Also, 1 day and 3 days after the first waterlogging treatment, both GB1126 and GB1129 that underwent waterlogging treatment displayed an increase in protein content in leaves compared with control. However, there was no significant difference in protein content in leaves between T1, T2, and C recorded 1 day after the second waterlogging treatment. However, 3 days after the second waterlogging treatment in genotype NJ, there was a significant decrease in protein content of T2 plant compared with C and an even more pronounced decrease in protein content of T1 plants compared with C (**Figure 5A**). Also, in the research by Seymen (2021), the protein content decreased with the exposure to the waterlogging stress.

In the stage of full ripeness of fruits on the first truss, both GB1126 and NJ showed an increase in protein content in leaves in both treatments (T1 and T2) compared with control, but in GB1129, there was an increase in protein content in leaves only for T2, whereas T1 had no significant difference compared with control (**Figure 5B**). In fruit samples, there were no significant differences between treatments and controls in protein content, except only between C and T1 in GB1129, where protein content was significantly higher in T1 compared with C and T2 (**Figure 5C**). This is consistent with the research by Niu et al. (2023), wherein one tomato genotype priming with waterlogging induced significantly lower protein content compared with plants that underwent only one waterlogging treatment.

### 3.4 The activity of class III peroxidase

Induction of POX activity is widely accepted as an indicator of abiotic and biotic stress response in plants (Veljović Jovanović et al., 2018). An increase in the activity of POX and polyphenol content could potentially mitigate the cellular damage caused by flooding (Lukić et al., 2021). Fruit samples of genotypes GB1126 and GB1129 grown in the controlled conditions exhibited the highest activity for POX and polyphenol oxidase, enzymes that have role in the defense responses against various stresses, in research where 10 genotypes from the Gene Bank of the Republic of Srpska were compared (Rašeta et al., 2022a).

**Figure 6:**
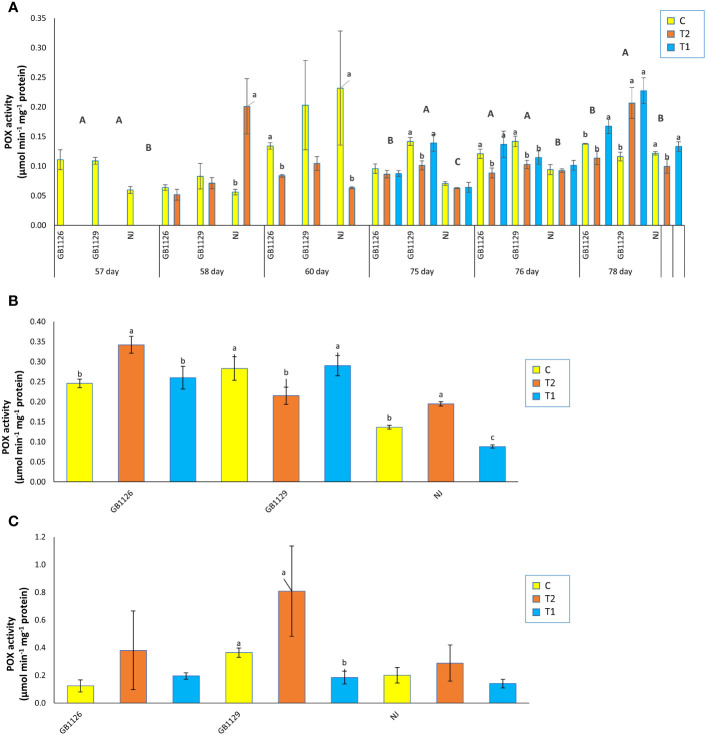
The activity of class III peroxidase (POX) in leaves prior to, 1 day after and 3 days after the 1^st^ and 2^nd^ waterlogging **(A)**, in leaves at the stage of full ripeness of the fruits on the 1^st^ truss **(B)** and in fruits **(C)** of tomato genotypes GB1126, GB1129 and NJ. Data represent mean ± SEM and was analyzed by ANOVA (between treatments (a, b and c) and between genotypes (A, B and C), where different letters indicate statistical significance) followed by post hoc Tukey's test. C-control; T1 - one waterlogging treatment; T2 - two waterlogging treatments.

There was no significant difference between GB1126 and GB1129 in POX activity in leaves prior to the first waterlogging treatment (**Figure 6A**). Next, in the stage of 1 day after the first waterlogging, genotype NJ showed a significant increase in POX activity in treated plants compared with control. This is in accordance with the other authors who observed increased POX activity due to waterlogging (Rasheed et al., 2018; Hao et al., 2019; Seymen, 2021). However, in leaves 3 days after the first waterlogging, both genotypes GB1126 and NJ showed a significant decrease in POX activity in T2 compared with C. Yin et al. (2023) suggested that waterlogging stress induced a significant increase in POX activity in some tomato genotypes and at the same time a significant decrease in other genotypes. Prior to the second waterlogging, there was a significant difference in POX activity among three genotypes. One day after the second waterlogging, genotype GB1126 exhibited a decrease in POX activity in T2 compared with C whereas GB1129 exhibited a decrease in POX activity in both T1 and T2 compared with C. Moreover, 3 days after the second waterlogging, genotype GB1126 showed a significant increase in POX activity in T1 compared with C, whereas T2 did not differ significantly from C, which aligns with the previous research by Niu et al. (2023), where in one tomato genotype priming with waterlogging induced significantly lower POX activity compared with plants that underwent only one waterlogging treatment. At the same stage, genotype GB1129 showed a significant increase in POX activity in both T1 and T2 compared with C whereas genotype NJ showed a significant decrease in POX activity in T1 compared with C (**Figure 6A**).

In the stage of full ripeness of fruits on the first truss, genotype GB1126 showed an increase in POX activity in T2 compared with C, genotype GB1129 showed a decrease in POX activity in T2 compared with C, and genotype NJ showed an increase in POX activity in T2 and a decrease in POX activity in T1 compared with C (**Figure 6B**). In fruit samples, there were no significant differences between treatments and controls in POX activity, except only between C and T1 in GB1129, which could suggest that waterlogging priming induced decreased POX activity due to waterlogging stress memory (**Figure 6C**).

### 3.5 Phenolic profiling

#### 3.5.1 Leaf and fruit-specific metabolic profiling

The effects of waterlogging on secondary metabolites of most commercial crops including tomato have not been widely addressed in the literature (Coutinho et al., 2018). Within the present study, extraction of samples was performed in 96% methanol, which was reported to be efficient in extracting the phenolic compounds in fruits of *Solanum* species (Milutinović et al., 2023). Using the targeted metabolomic approach, three phenolic acids (caffeic acid and two isomers of caffeoylquinic acid; syn. chlorogenic acid: 3-*O*-caffeoylquinic acid and 5-*O*-caffeoylquinic acid) and six flavonoids (quercetin-3-*O*-glucoside, kaempferol-3-*O*-glucoside, rutin, naringenin, naringin, and eriodyctiol) were quantified in the leaves of analyzed tomato genotypes. In fruits, two more flavonoid aglycones (luteolin and apigenin) were present in significant amounts (see data availability statement). Three compounds (5-*O*-caffeoylquinic acid, rutin, and quercetin 3-*O*-glucoside) are characteristic of all tested leaf and fruit tomato samples; namely, they were found in all samples in all repetitions. Kaempferol 3-*O*-glucoside was quantified in all leaf samples, whereas in the fruit, it was present only in T1 plants of genotype GB1129. This follows the research by Martínez-Valverde et al. (2002), where only two out of nine tomato genotypes contained kaempferol 3-*O*-glucoside in the fruit.

Caffeic acid, 3-*O*-caffeoylquinic acid, 5-*O*-caffeoylquinic acid, kaempferol 3-*O*-glucoside, rutin, naringenin, and naringin are among 48 phenolic compounds found at Metabolome Tomato Database (MoTo DB), which consists of phenolic compounds reported to be present in the tomato fruit extracts (Moco et al., 2006). More recently, Tohge and Fernie (2015) provided an inventory of metabolites reported in tomato consisting of 122 flavonoids and 56 hydroxycinnamates (total of 178 phenolics), which includes all phenolic compounds found in our research except luteolin. Moreover, there are several studies in which the metabolites of tomato fruit have been investigated by using LC/MS methods (Moco et al., 2007; Iijima et al., 2008; Alseekh et al., 2015). On the other hand, there are yet few studies investigating different tomato genotypes for their metabolite contents (Siracusa et al., 2013; Baldina et al., 2016; Di Paola Naranjo et al., 2016).

Regarding hydroxycinnamates, MS^2^ fragments at *m/z* 107 and 135 are identified as caffeic acid, which has been previously reported in tomato by Tohge and Fernie (2015) and Milutinović et al. (2023). Two isomers of caffeoylquinic acid; syn. chlorogenic acid: 3-*O*-caffeoylquinic acid and 5-*O*-caffeoylquinic acid (MS^2^ fragments at *m/z* 191) have been identified in tomato by Baldina et al. (2016) and Di Paola Naranjo et al. (2016). The major phenolic compounds in fruits of all three genotypes were 5-*O*-caffeoylquinic acid, which reached the amounts up to 260 µg g^−1^ DW. Regarding flavonoids, quercetin-3-*O*-glucoside, kaempferol-3-*O*-glucoside, and naringenin have been reported in tomato by Wu et al. (2013) and Martínez-Valverde et al. (2002). In tomato fruit, the presence of rutin, naringin, and eriodictyol was identified by Anton et al. (2017). Apigenin has been reported in tomato by Tohge and Fernie (2015), and luteolin has been reported in the Solanaceae family (Oertel et al., 2017).

Phenolic compounds have been extensively characterized in tomato varieties from different countries (Baldina et al., 2016; Di Paola Naranjo et al., 2016). The chemical composition of tomato fruits can vary according to the cultivar, cultivation conditions, handling, and storage methods (Barros et al., 2012). Although some previous publications describe the morphology and total phenolic and total flavonoid content of the two local varieties from the Gene Bank of the Republic of Srpska (Rašeta et al., 2022b, 2023), this study describes for the first time the quantitative content of targeted phenolic compounds in leaves and fruits of GB1126 and GB1129 genotypes.

#### 3.5.2 Developmentally regulated profiles of phenolic compounds in tomato leaves and fruits as influenced by the genotype

**Figure 7:**
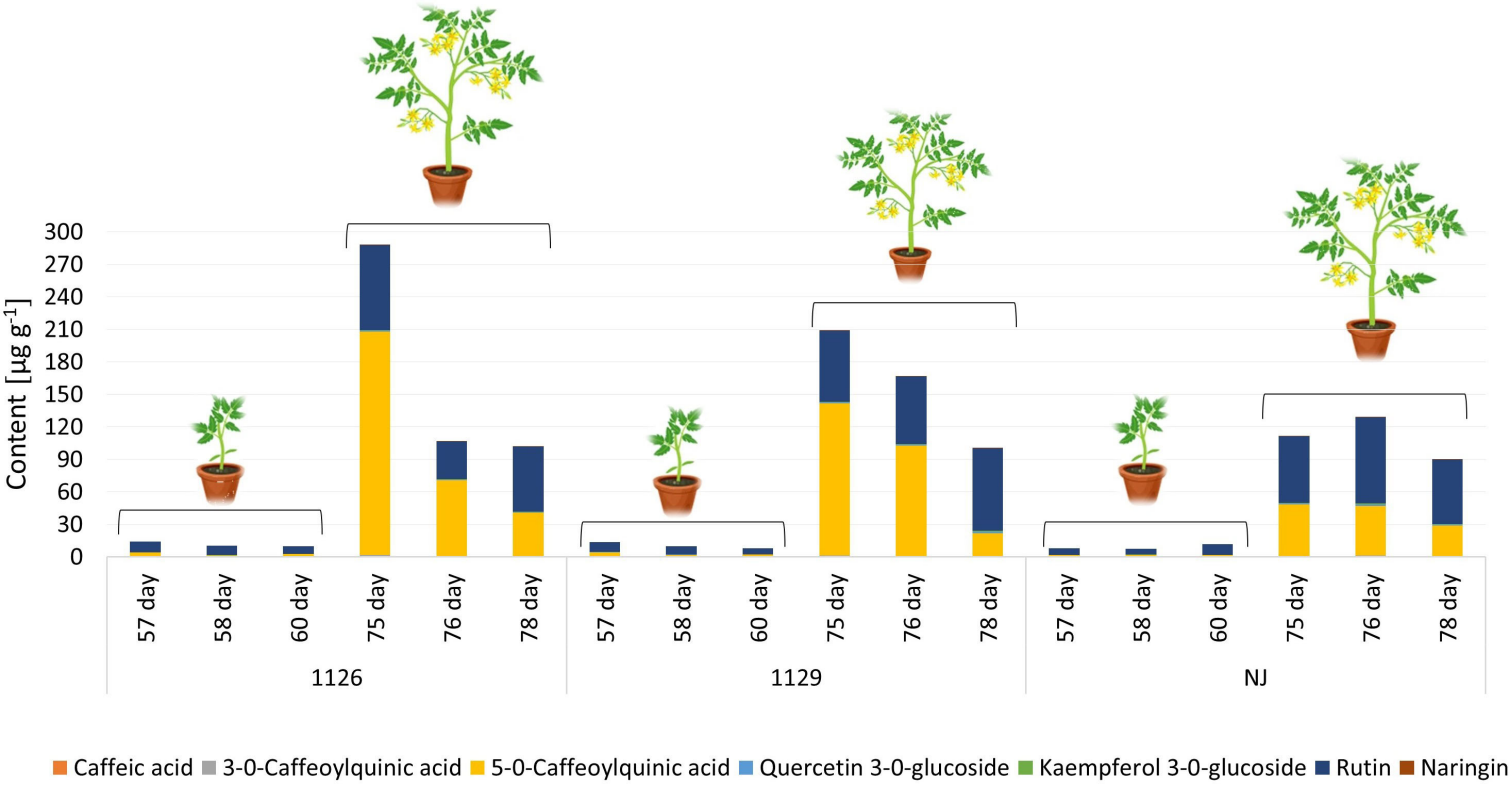
The content of major phenolic compounds in leaves of tomato varieties GB01126, GB01129 and NJ at seedling stage (57-60 day) and at full flowering stage (75-78 day). Data represent mean ± SEM and was analyzed by ANOVA followed by post hoc Tukey’s test (please refer to **Supplementary Table 3**).

In the leaf samples collected prior to, 1 day, and 3 days after the first and second waterlogging, seven targeted phenolic compounds were quantified: caffeic acid, 3-*O*-caffeoylquinic acid, 5-*O*-caffeoylquinic acid, quercetin-3-*O*-glucoside, kaempferol-3-*O*-glucoside, rutin, and naringenin. The content of major phenolic compounds in leaves varied significantly according to genotype (**Figure 7**; **Supplementary Table 3**). The literature also confirms cultivar dependence in phenolic profiles of tomato (Barros et al., 2012; Anton et al., 2017). Major phenolics in all genotypes were 5-*O*-caffeoylquinic acid from the group of phenolic acids and flavonoid rutin. According to the literature, the main phenolic compound in tomato is chlorogenic acid (5-*O*-caffeoylquinic acid), followed by naringenin, quercetin, rutin, and kaempferol (Martínez-Valverde et al., 2002; Vallverdú-Queralt et al., 2010; Rosa-Martínez et al., 2023), which is similar to our results. Leaves collected at the full flowering stage, from 75 day-old plants, had higher amounts of phenolic compounds than leaves at the stage of seedlings. This trend was also observed for the GB1129 genotype. The highest amounts of phenolics in leaves were detected in genotype GB1126, which was followed by genotype GB1129 (**Figure 7**). A significant decrease in the amounts of targeted phenolics was observed for 76- and 77-day-old plants. As for leaves of NJ genotype, the highest amount of phenolics were reached for 75-day-old plants, and the amount of phenolic was not changed in the following 2 days.

**Figure 8:**
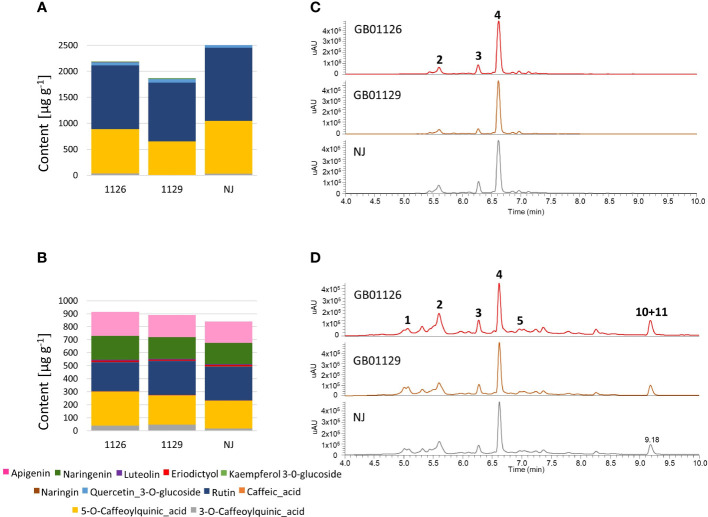
The content of major phenolic compounds in leaves **(A)** and fruits **(B)** of GB01126, GB01129 and NJ genotypes at the stage of fruit ripeness; and UHPLC/DAD chromatograms at λ=254 nm of corresponding methanol extracts in leaves **(C)** and fruits **(D)**. **1** – 3-*O*-Caffeoylquinic acid; **2** – 5-*O*-Caffeoylquinic acid; **3** – Caffeic acid; **4** – Rutin; **5** – Quercetin 3-*O*-glucoside; **10** – Naringenin; **11** – Apigenin. Data represent mean ± SEM and was analyzed by ANOVA followed by post hoc Tukey's test (please refer to **Supplementary Tables 4**, **5**).

In the leaf samples from the tomato plants in the stage of full ripeness of fruits on the first truss (in 132-day-old plants), eight targeted phenolic compounds were quantified: 3-*O*-caffeoylquinic acid, 5-*O*-caffeoylquinic acid, quercetin-3-*O*-glucoside, kaempferol-3-*O*-glucoside, rutin, naringenin, naringin, and eriodyctiol (**Figures 8A, C**, **Supplementary Tables 4**, **5**). Caffeic acid was not identified at this stage. The NJ genotype displayed the highest content of targeted phenolics in leaves, whereas genotype GB1129 generally showed the lowest content of phenolics. The differences in phenolic profiles between leaf samples during different stages were observed, which is in line with research by Dadáková et al. (2020).

In the fruit samples, 11 targeted phenolic compounds were identified and quantified: caffeic acid, 3-*O*-caffeoylquinic acid, 5-*O*-caffeoylquinic acid, quercetin-3-*O*-glucoside, kaempferol-3-*O*-glucoside, rutin, naringenin, luteolin, and apigenin (**Figures 8B, D**). When the content of phenolics in fruits was compared between genotypes, it was observed that GB1126 contained a slightly higher content of targeted compounds than GB1129 and NJ. Taking into account the smaller fruit size in GB1126, when compared with GB1129 and NJ, as well as the fact that phenolics are mostly accumulated in the skin of the tomato fruits (Quinet et al., 2019; Tamasi et al., 2019), it could be concluded that differences in phenolic profiles might be, at least partially, attributed to the fruit skin to volume ratio. According to Marsic et al. (2011), higher levels of total phenolic content in smaller tomatoes, compared with cultivars with larger fruits, are due to higher skin to volume ratio in fruits of these varieties, and this determines the phenolic content, particularly of flavonols. In fruits, these compounds tend to accumulate in dermal tissues where they play a potential role in protection against UV radiation, as attractants in fruit dispersal or as defense chemicals against pathogens and predators (Tsao and McCallum, 2009).

The differences in phenolic profiles between leaf and fruit samples were observed. The literature also confirms organ dependence in phenolic profiles of tomato, with roots being completely differentiated from leaves and stems (Larbat et al., 2012).

#### 3.5.3 Genotype-dependent phenolic profiles in leaves of fully-flowering plants as influenced by waterlogging treatment

**Figure 9:**
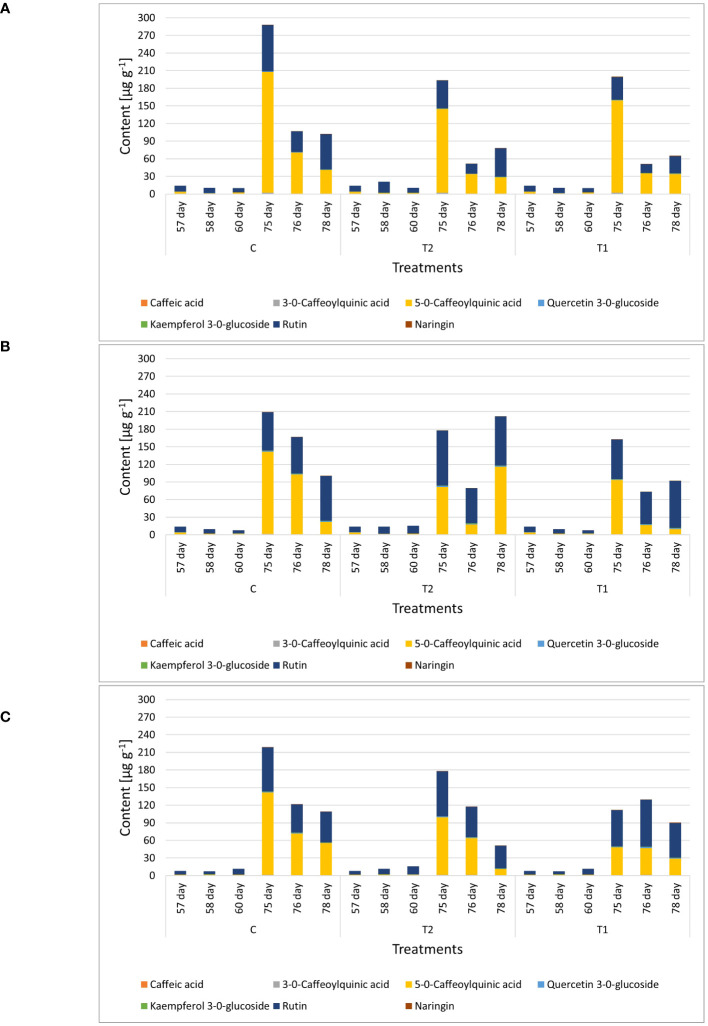
Genotype-dependent phenolic profiles in leaves of GB01126 **(A)**, GB01129 **(B)**, and NJ **(C)** genotypes at seedling and full-flowering stage as influenced by waterlogging. Data represent mean ± SEM and was analyzed by ANOVA followed by post hoc Tukey's test (please refer to **Supplementary Tables 6**-**8**). C - control; T1 - one waterlogging treatment; T2 - two waterlogging treatments.

**Figure 10:**
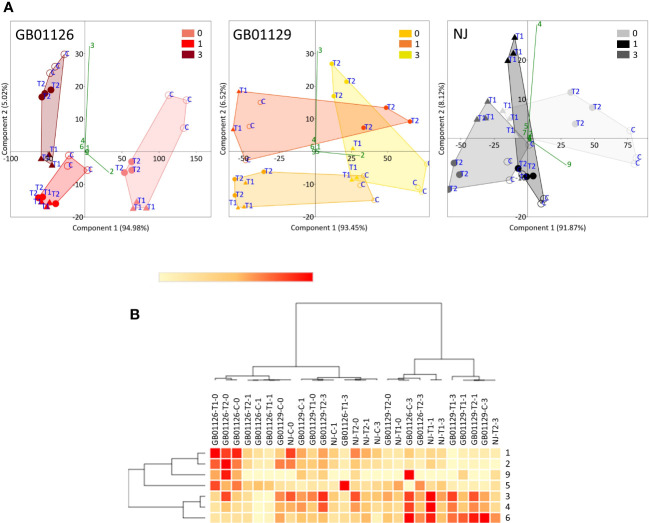
Genotype-dependent phenolic profiles in leaves of fully-flowering tomato plants prior (0), 1 day after (1) and 3 days after (3) the 2^nd^ waterlogging treatment: **(A)** PCA plots, independently for GB01126, GB01129, and NJ, indicating differences between the treatments. **(B)** Heatmap of the scaled quantitative data of targeted phenolics, with the samples (both columns and rows) arranged according to the HCA (Pearson method of cluster agglomeration). Intensity of red and yellow color indicate the amounts of targeted compounds in samples, with red color representing the max values and yellow color the min values recorded for individual metabolite, as indicated in the color scale. **1**- 3-*O*-Caffeoylquinic_acid, **2**- 5-*O*-Caffeoylquinic_acid, **3**- Rutin, **4**- Quercetin_3-O-glucoside, **5**- Naringin, **6**- Kaempferol_3-O-glucoside, **7**- Eriodictyol, **8**- Naringenin, **9**- Caffeic_acid, **10**- Luteolin, **11**- Apigenin.

Leaf samples of genotype GB1126 grown under control growth regime had higher content of phenolics before the second waterlogging (75-day-old plants) compared with plants previously exposed to first waterlogging treatment (**Figure 9A**, **Supplementary Table 6**). Thus, the first waterlogging treatment at the seedling stage resulted in the decrease of targeted phenolics in leaves at the flowering stage of plant growth. One day after the second waterlogging, a significant decrease in the content of phenolics can be observed on all treatments, which could be assigned primarily to the decrease in 5-*O*-caffeoylquinic acid and rutin. Three days after the second waterlogging, in T1 and T2 plants, we can observe a slight increase in rutin content. A PCA plot for genotype GB1126, with PC1 and PC2 describing 94.98% and 5.00% of the total variability, respectively, clearly separates leaf samples prior to the second waterlogging treatment and those after the treatment along the PC1 (**Figure 10A**). Slight separation between phenolics in leaves of T1-treated plants on the one hand, and control and T2-treated plants on the other, can be observed 3 days after the second waterlogging, along the PC2, with T2 samples being more similar to non-treated samples than T1. This might imply that T2 plants exhibited plant stress memory. Li et al. (2011) reported that flooding pretreatment during vegetative growth improves tolerance to flooding in reproductive stage in wheat. However, the epigenetic regulatory pathways of flooding memory and the involvement of transcriptional memory in the flooding recovery period are not well investigated in the literature (Liu et al., 2021). Major contributors to the diversification between samples along component 1 are 5-*O*-caffeoylquinic acid and rutin, the latter being also the most significant contributor to the diversification of samples along the PC2.

Similar phenolic profiles in leaves are observed for the GB1129 genotype. After an initial decrease in phenolic content 1 day after the second waterlogging, T1 and T2 leaves experience an even more pronounced increase in 5-*O*-caffeoylquinic acid and rutin content 3 days after the second waterlogging (**Figure 9B**, **Supplementary Table 7**). In the GB1129 genotype, the PCA plot depictures clear separation along PC1 between non-treated and treated plants (both T1 and T2) 1 day after the second waterlogging (**Figure 10A**). The content of 5-*O*-caffeoylquinic acid is significantly decreased in T1 and T2 plants 1 day after the second waterlogging treatment. Differences in phenolic profile in leaves between T1 and T2 plants are visible 3 days after the second waterlogging, when T2 plants experience the pronounced increase in 5-*O*-caffeoylquinic acid content. The major contributor to the diversification between samples along PC1, which describes 93.45% of the total variability, is 5-*O*-caffeoylquinic acid (**Figure 10A**). Rutin is the major contributor to the diversification between samples along the PC2, which contributes with 6.52% to the total variance.

For the NJ genotype, the decrease in phenolic content 3 days after the second waterlogging was more pronounced in T2 plants (**Figure 9C**, **Supplementary Table 8**), which can preferentially be attributed to the decrease in the content of 5-*O*-caffeoylquinic acid. PCA plots show clear diversification between samples before and after the second waterlogging (**Figure 10A**). Moreover, 3 days after the second waterlogging, T2-treated plants were more similar to control plants than T1 plants. Major contributors to the diversification between samples along PC1, which describes 91.87% of the total variability, are 5-*O*-caffeoylquinic acid and rutin. Rutin also significantly contributes to the separation of samples along the PC2, which explains 8.12% of the total variability.

This is also visible on HCA plot constructed based on the Pearson algorithm (**Figure 10B**). Genotypes GB1129 and NJ are more similar in words of phenolic profiles. When correlation analysis was performed (data not presented), it was clear that the content of three phenolic acids (5-*O*-caffeoylquinic acid, 3-*O*-caffeoylquinic acid, and caffeic acid) is significantly positively correlated. Rutin was significantly positively correlated with quercetin-3-*O*-glucoside.

#### 3.5.4 Leaf and fruit phenolic profiles in plants at the stage of full ripeness of fruits on the first truss as influenced by waterlogging treatments

We further examined the changes in the profiles of major phenolics in leaves and fruits of the three tomato genotypes in the stage of full ripeness of fruits on the first truss, as influenced by waterlogging treatments.

**Figure 11:**
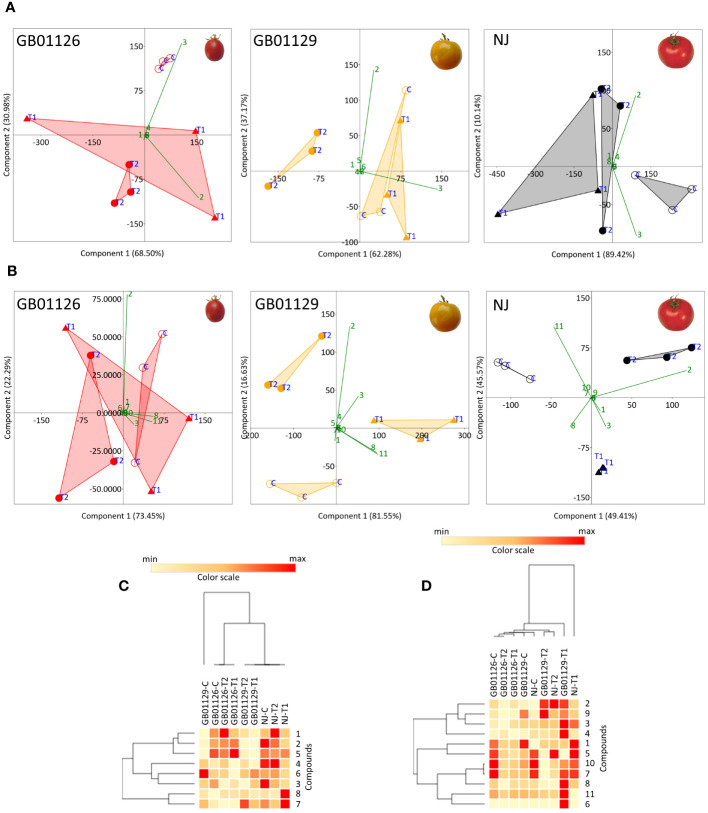
PCA plots of phenolic profiles in leaves **(A)** and fruits **(B)** of GB01126, GB01129, and NJ tomato genotypes at the stage of full ripeness as influenced by waterlogging treatments. Compounds are labeled with numbers, as explained in abbreviations. Heatmap of the scaled quantitative data of targeted phenolics in the leaves **(C)** and fruits **(D)** at the stage of full ripeness, with the samples (both columns and rows) arranged according to the HCA (Spearman method of cluster agglomeration). Intensity of red and yellow color indicate the amounts of targeted compounds in samples, with red color representing the max values and yellow color the min values recorded for individual metabolite, as indicated in the color scale. **1**- 3-O-Caffeoylquinic_acid, **2**- 5-O-Caffeoylquinic_acid, **3**- Rutin, **4**- Quercetin_3-O-glucoside, **5**- Naringin, **6**- Kaempferol_3-O-glucoside, **7**- Eriodictyol, **8**- Naringenin, **9**- Caffeic acid, **10**- Luteolin, **11**- Apigenin. * C-control; T1 - one waterlogging treatment; T2 - two waterlogging treatments.

Phenolic profiles of leaves in GB1126 control were clearly diversified from the leaves of T2-treated plants along PC1, which explained 68.50% of the total variability (**Figure 11A**). The phenolic profile of leaves of T1-treated plants was variable, with no clear relations with control and T2-treated plants. Similarly, in GB1129, the control and T2-treated plants were more different in words of phenolic profiles of leaves, as they diversified along PC1 (62.28%). The phenolic profile of leaves of T1-treated plants was more similar to that of the control group of plants (**Figure 11A**). A similar trend was observed also for NJ leaves, as the control group was clearly diversified from T2-treated plants along PC1 (89.42%). In all these cases, 5-*O*-caffeoylquinic acid and rutin were the major factors determining the separation of samples along PC1 (**Figure 11A**). These two compounds are highlighted here as useful markers to evaluate the waterlogging tolerance of tomato varieties, as their content is significantly decreased in leaves under waterlogging treatments, especially following repeated waterlogging. Similar trends were observed when HCA was performed on the quantified data (**Figure 11B**). Following waterlogging treatments, phenolic profiles of leaves were less altered in GB1126 than GB1129 and NJ.

In fruits of GB1126, GB1129, and NJ, the opposite trend was observed, as the content of 5-*O*-caffeoylquinic acid, 3-*O*-caffeoylquinic acid, and rutin increased following the waterlogging treatments. On the other hand, the content of apigenin and naringenin decreased at T1 and T2 treatments. PCA plots showed that waterlogging treatments induced changes in phenolic profiles of fruits of GB1126, GB1129, and NJ, which were more pronounced in NJ and GB1129 (**Figure 11C**). In GB1126, significant differences were observed only between fruits of control and T2 plants (**Figure 11C**). In all three genotypes, 5-*O*-caffeoylquinic acid, rutin, apigenin, and naringenin were the major factors contributing to the diversification of samples in the PCA plot. This can also be seen from the HCA plot (**Figure 11D**).

The phenolic profiles of leaves and fruits of GB1126 were the least susceptible to changes in response to waterlogging, and repeated waterlogging treatments, indicating that this variety was more tolerant to than GB1129 and NJ. Ngumbi et al. (2022) also reported a difference between tomato genotypes in the production of secondary metabolites including phenylpropanoids and terpenoids upon stress associated with flooding.

### 3.6 Correlation between analyzed parameters

Oxidative stress plays an important role in waterlogging-stressed plants and the protection from oxidative damage results, at least in part, through the maintenance of increased antioxidative enzyme activity and non-enzymatic antioxidants (Šola and Stić, 2021; Yin et al., 2023). Tomato plants that were subjected to priming under waterlogging stress (repeated waterlogging stress) showed lower peroxidase activity but higher hydrogen peroxidase content compared with plants that were subjected only to waterlogging (Niu et al., 2023). In our research, we have made a sum of all of the phenolics detected, in order to do correlations with other parameters.

We found that the oxidative parameters (H_2_O_2_ and MDA contents) showed a substantially significantly positive correlation with phenolic compounds (correlation coefficient = 0.862*** and 0.573***) and proteins (correlation coefficient = 0.912*** and 0.645***) (**Table 3**). A positive correlation between oxidative parameters and the concentration of proteins and phenolic compounds may indicate that by increasing the concentration of H_2_O_2_ and MDA, the concentration of proteins and phenolic compounds also increases, that is, their synthesis is induced. Also, oxidative parameters were substantially significantly correlated among them (H_2_O_2_ and MDA content) (correlation coefficient = 0.760***). One of the sources of MDA in the cell is the higher concentration of H_2_O_2_. The positive correlation between these parameters indicates the existence of other sources of MDA in tomato cells. In the waterlogging-stress study, oxidative stress markers such as H_2_O_2_ and MDA were negatively correlated with most of the antioxidant enzymes (Anee et al., 2019).

**Table 3:** Correlation analysis of all the measured parameters (Pearson’s correlation coefficients: **P < 0.01, ***P < 0.001); POX, class III peroxidase; H_2_O_2_, hydrogen peroxidase; MDA, malondialdehyde.

	Phenols	Proteins	POX	H_2_O_2_	MDA
Phenols	Phenols	0.735	0.432	0.862	0.573
Proteins	***	Proteins	0.040	0.912	0.645
POX	**		POX	0.215	0.049
H_2_O_2_	***	***		H_2_O_2_	0.760
MDA	***	***		***	MDA

Meanwhile, POX showed no significant correlation with other parameters, except the significant correlation with phenolic compounds (correlation coefficient = 0.432**). Lukić et al. (2021) found that one maize genotype exhibited a positive correlation between POX and polyphenols, whereas other maize genotypes showed a negative correlation between those parameters. They suggested that in the genotype with a positive correlation, polyphenols likely played an antioxidant role by directly neutralizing ROS. Moreover, in our research, polyphenols exhibited a substantially significantly positive correlation with proteins (correlation coefficient = 0.735***).

## 4 Conclusion

The establishment of selection criteria for waterlogging-tolerant genotypes is critical for the expansion of cultivation, particularly in areas with frequent and high rainfall, which is the case in the northern parts of the Republic of Srpska (Bosnia and Herzegovina). An ideal waterlogging-tolerant tomato cultivar should not only survive waterlogging but also rapidly recover to the control level. According to waterlogging tolerance tests, genotype GB1126 showed the best tolerance to waterlogging stress, as it displayed a lower percentage of wilted plants, lower YLP, and higher ARF percentage, when compared with GB1129 and NJ. This conclusion was supported by the analyses of other parameters.

Oxidative parameters (H_2_O_2_ and MDA) exhibited an increase in content in leaves of tomato plants that underwent waterlogging stress compared with control plants. However, genotype GB1126 exhibited a significant decrease in oxidative parameters at some stages, implying certain waterlogging tolerance. Also, it could be argued that waterlogging priming induced waterlogging memory associated with H_2_O_2_ in tomato when stress reoccurred. Differentiation between phenolic profiles in leaves of non-treated and waterlogging-treated plants can be observed shortly after the second treatment. Both genotypes GB1129 and GB1126 which went through the repeated waterlogging treatments display a prominent increase in phenolic content 3 days after the second waterlogging treatment. This trend is also observed in genotypes GB1129 and GB1126, which went through waterlogging treatment only once, but was less pronounced. This might indicate that T2 plants exhibited plant stress memory. Waterlogging priming can induce stress memory by adjusting the content of phenolics in tissues, which are of significant importance for maintaining the redox homeostasis, and thus for the alleviation of the damage of ROS on tomato when waterlogging reoccurs. Observed changes could be primarily ascribed to the prominent changes in the content of the two major phenolic compounds in leaves, 5-*O*-caffeoylquinic acid and rutin. The decrease in the content of these compounds in leaves of tomato is the obvious symptom of waterlogging stress.

Phenolic compounds in leaves and fruits of tomato varieties show a sensitive response to waterlogging stress, which provides a tool for evaluating the stress tolerance in different tomato varieties. Plant selection for increased tolerance to stress factors requires parameters with high sensitivity, as well as fast and inexpensive measurements, and the content of phenolics in tomato tissues can be efficiently evaluated with low costs. Among analyzed genotypes, GB1126 is the most efficient in maintaining the phenolic profiles of leaves and fruits, and thus of the nutritive and organoleptic qualities of fruits following the exposure to waterlogging.

Taking into account the results of the present study, GB1126 is a good candidate for extensive cultivation, as it displays significant tolerance to waterlogging stress, which includes the ability to maintain physiological state of leaves, and to improve the oxygen availability directly from the air by adventitious root formation. Research demonstrated that waterlogging priming can trigger stress memory, enhancing their tolerance to subsequent waterlogging stress, but this effect was not observed in all growth stages. The waterlogging priming was the most emphasized in fruit samples at the stage of full ripeness of fruits on the first truss.

The capacity of genotypes to adapt to stressful conditions entails the activation of different response mechanisms, thus affecting processes at the morphological, physiological, or molecular levels. In the future, in order to deeply and comprehensively understand the metabolite disparity of tomato genotypes, multi-omics data should be integrated with metabolome data.

## Data availability statement

The original contributions presented in the study are included in the article/**Supplementary Material**. Further inquiries can be directed to the corresponding authors.

## Author contributions

SU: Conceptualization, Data curation, Formal analysis, Investigation, Writing – original draft. BK: Conceptualization, Formal analysis, Investigation, Supervision, Writing – review & editing. IM: Conceptualization, Supervision, Writing – review & editing. UG: Data curation, Formal analysis, Investigation, Methodology, Writing – review & editing. MM: Data curation, Formal analysis, Investigation, Methodology, Writing – review & editing. MA: Conceptualization, Formal analysis, Methodology, Writing – review & editing. DM: Conceptualization, Data curation, Funding acquisition, Methodology, Resources, Supervision, Writing – review & editing.

## References

[B1] Ahsan N. Lee D. G. Lee S. H. Lee K. W. Bahk J. D. Lee B. H. (2007). A proteomic screen and identification of waterlogging-regulated proteins in tomato roots. Plant Soil 295, 37–51. doi: 10.1007/s11104-007-9258-9

[B2] Alseekh S. Tohge T. Wendenberg R. Scossa F. Omranian N. Li J.. (2015). Identification and mode of inheritance of quantitative trait loci for secondary metabolite abundance in tomato. Plant Cell 27, 485–512. doi: 10.1105/tpc.114.132266 25770107 PMC4558650

[B3] Anee T. I. Nahar K. Rahman A. Mahmud J. A. Bhuiyan T. F. Alam M. U.. (2019). Oxidative damage and antioxidant defence in Sesamum indicum after different waterlogging durations. Plants 8, 196. doi: 10.3390/plants8070196 31261970 PMC6681296

[B4] Anton D. Bender I. Kaart T. Roasto M. Heinonen M. Luik A.. (2017). Changes in polyphenols contents and antioxidant capacities of organically and conventionally cultivated tomato (*Solanum lycopersicum* L.) fruits during ripening. Int. J. Analytical Chem. 2017, 1–11. doi: 10.1155/2017/2367453 PMC5463128 28630627

[B5] Ashraf M. A. Ashraf M. Shahbaz M. (2012). Growth stage-based modulation in antioxidant defense system and proline accumulation in two hexaploid wheat (Triticum aestivum L.) cultivars differing in salinity tolerance. Flora-Morphol. Distribution Funct. Ecol. Plants 207, 388–397. doi: 10.1016/j.flora.2012.03.004

[B6] Baldina S. Picarella M. E. Troise A. D. Pucci A. Ruggieri V. Ferracane R.. (2016). Metabolite profiling of Italian tomato landraces with different fruit types. Front. Plant Sci. 7. doi: 10.3389/fpls.2016.00664 PMC4872001 27242865

[B7] Barros L. Dueñas M. Pinela J. Carvalho A. M. Buelga C. S. Ferreira I. C. (2012). Characterization and quantification of phenolic compounds in four tomato (*Lycopersicon esculentum* L.) farmers’ varieties in northeastern Portugal homegardens. Plant Foods Hum. Nutr. 67, 229–234. doi: 10.1007/s11130-012-0307-z 22922837

[B8] Bhatt R. M. Upreti K. K. Divya M. H. Bhat S. Pavithra C. B. Sadashiva A. T. (2015). Interspecific grafting to enhance physiological resilience to flooding stress in tomato (*Solanum lycopersicum* L.). Scientia Hortic. 182, 8–17. doi: 10.1016/j.scienta.2014.10.043

[B9] Broad Institute (2023) Morpheus software. Available online at: https://software.broadinstitute.org/morpheus (Accessed October 20, 2023).

[B10] Cotrozzi L. Lorenzini G. Nali C. Pisuttu C. Pampana S. Pellegrini E. (2021). Transient waterlogging events impair shoot and root physiology and reduce grain yield of durum wheat cultivars. Plants 10, 2357. doi: 10.3390/plants10112357 34834720 PMC8625979

[B11] Coutinho I. D. Henning L. M. M. Döpp S. A. Nepomuceno A. Moraes L. A. C. Marcolino-Gomes J.. (2018). Flooded soybean metabolomic analysis reveals important primary and secondary metabolites involved in the hypoxia stress response and tolerance. Environ. Exp. Bot. 153, 176–187. doi: 10.1016/j.envexpbot.2018.05.018

[B12] Crawford R. M. M. (1982). “Physiological responses to flooding,” in Physiological Plant Ecology II. Encyclopedia of Plant Physiology, vol. 12/B . Eds. Lange O. L. Nobel P. S. Osmond C. B. Ziegler H. (Springer, Berlin, Heidelberg). doi: 10.1007/978-3-642-68150-9_15

[B13] Dadáková K. Heinrichová T. Lochman J. Kašparovský T. (2020). Production of defense phenolics in tomato leaves of different age. Molecules 25, 4952. doi: 10.3390/molecules25214952 33114660 PMC7663536

[B14] Di Paola Naranjo R. D. Otaiza S. Saragusti A. C. Baroni V. Carranza A. D. V. Peralta I. E.. (2016). Hydrophilic antioxidants from Andean tomato landraces assessed by their bioactivities in *vitro* and in *vivo* . Food Chem. 206, 146–155. doi: 10.1016/j.foodchem.2016.03.027 27041310

[B15] Ezin V. Pena R. D. L. Ahanchede A. (2010). Flooding tolerance of tomato genotypes during vegetative and reproductive stages. Braz. J. Plant Physiol. 22, 131–142. doi: 10.1590/S1677-04202010000200007

[B16] Ezin V. Vodounon C. A. de la Peña R. Ahanchede A. Handa A. K. (2012). Gene expression and phenotypic characterization of flooding tolerance in tomato. J. Evolutionary Biol. Res. 4, 59–65. doi: 10.5897/JEBR12.009

[B17] Food and Agriculture Organization (2021) Tomato. Available online at: https://www.fao.org/faostat/en/#data/QCL (Accessed October 22, 2023).

[B18] Hammer Ø. Harper D. A. (2001). Past: paleontological statistics software package for education and data anlysis. Palaeontologia Electronica 4, 1.

[B19] Hao S. Cao H. Wang H. Pan X. (2019). The physiological responses of tomato to water stress and re-water in different growth periods. Scientia Hortic. 249, 143–154. doi: 10.1016/j.scienta.2019.01.045

[B20] Heath R. L. Packer L. (1968). Photoperoxidation in isolated chloroplasts. I. Kinetics and stoichiometry of fatty acid peroxidation. Arch. Biochem. Biophys. 125, 189–198. doi: 10.1016/0003-9861(68)90654-1 5655425

[B21] Horchani F. Gallusci P. Baldet P. Cabasson C. Maucourt M. Rolin D.. (2008). Prolonged root hypoxia induces ammonium accumulation and decreases the nutritional quality of tomato fruits. J. Plant Physiol. 165, 1352–1359. doi: 10.1016/j.jplph.2007.10.016 18180072

[B22] Iijima Y. Suda K. Suzuki T. Aoki K. Shibata D. (2008). Metabolite profiling of chalcones and flavanones in tomato fruit. J. Japanese Soc. Hortic. Sci. 77, 94–102. doi: 10.2503/jjshs1.77.94

[B23] Insausti P. Gorjón S. (2013). Floods affect physiological and growth variables of peach trees (Prunus persica (L.) Batsch), as well as the postharvest behavior of fruits. Scientia Hortic. 152, 56–60. doi: 10.1016/j.scienta.2013.01.005

[B24] Irving L. J. Sheng Y. B. Woolley D. Matthew C. (2007). Physiological effects of waterlogging on two lucerne varieties grown under glasshouse conditions. J. Agron. Crop Sci. 193, 345–356. doi: 10.1111/j.1439-037X.2007.00277.x

[B25] Kukavica B. Mojović M. Vucinić Z. Maksimović V. Takahama U. Veljović-Jovanović S. (2009). Generation of hydroxyl radical in isolated pea root cell wall, and the role of cell wall-bound peroxidase, Mn-SOD and phenolics in their production. Plant Cell Physiol. 50, 304–317. doi: 10.1093/pcp/pcn199 19098072

[B26] Kumar P. Pal M. Joshi R. Sairam R. K. (2013). Yield, growth and physiological responses of mung bean [*Vigna radiata* (L.) Wilczek] genotypes to waterlogging at vegetative stage. Physiol. Mol. Biol. Plants 19, 209–220. doi: 10.1007/s12298-012-0153-3 24431488 PMC3656181

[B27] Larbat R. Le Bot J. Bourgaud F. Robin C. Adamowicz S. (2012). Organ-specific responses of tomato growth and phenolic metabolism to nitrate limitation. Plant Biol. 14, 760–769. doi: 10.1111/j.1438-8677.2012.00564.x 22372822

[B28] Li C. Jiang D. Wollenweber B. Li Y. Dai T. Cao W. (2011). Waterlogging priming during vegetative growth improves tolerance to waterlogging after anthesis in wheat. Plant Sci. 180, 672–678. doi: 10.1016/j.plantsci.2011.01.009 21421417

[B29] Liu H. Able A. J. Able J. A. (2021). Priming crops for the future: rewiring stress memory. Trends Plant Sci. 27, 699–716. doi: 10.1016/j.tplants.2021.11.015 34906381

[B30] Lowry O. H. Rosebrough N. J. Farr A. L. Randall R. J. (1951). Protein measurement with the Folin phenol reagent. J. Biol. Chem. 193, 265–275. doi: 10.1016/S0021-9258(19)52451-6 14907713

[B31] Lukić N. Trifković T. Kojić D. Kukavica B. (2021). Modulations of the antioxidants defence system in two maize hybrids during flooding stress. J. Plant Res. 134, 237–248. doi: 10.1007/s10265-021-01264-w 33591473

[B32] Marsic N. K. Gasperlin L. Abram V. Budic M. Vidrih R. (2011). Quality parameters and total phenolic content in tomato fruits regarding cultivar and microclimatic conditions. Turkish J. Agric. Forestry 35, 185–194. doi: 10.3906/tar-0910-499

[B33] Martínez-Valverde I. Periago M. J. Provan G. Chesson A. (2002). Phenolic compounds, lycopene and antioxidant activity in commercial varieties of tomato (*Lycopersicum esculentum*). J. Sci. Food Agric. 82, 323–330. doi: 10.1002/jsfa.1035

[B34] Meier U. (2001). Growth stages of mono and dicotyledonous plants. BBCH Monograph (Federal Biological Research Centre for Agriculture and Forestry).

[B35] Mellidou I. Koukounaras A. Kostas S. Patelou E. Kanellis A. K. (2021). Regulation of vitamin C accumulation for improved tomato fruit quality and alleviation of abiotic stress. Genes 12, 694. doi: 10.3390/genes12050694 34066421 PMC8148108

[B36] Michalak A. (2006). Phenolic compounds and their antioxidant activity in plants growing under heavy metal stress. Polish J. Environ. Stud. 15, 523–530.

[B37] Milutinović M. Nakarada Đ. Božunović J. Todorović M. Gašić U. Živković S.. (2023). *Solanum dulcamara* L. Berries: A convenient model system to study redox processes in relation to fruit ripening. Antioxidants 12, 346. 36829905 10.3390/antiox12020346 PMC9952312

[B38] Moco S. Bino R. J. Vorst O. Verhoeven H. A. de Groot J. van Beek T. A.. (2006). A liquid chromatography-mass spectrometry-based metabolome database for tomato. Plant Physiol. 141, 1205–1218. doi: 10.1104/pp.106.078428 16896233 PMC1533921

[B39] Moco S. Capanoglu E. Tikunov Y. Bino R. J. Boyacioglu D. Hall R. D.. (2007). Tissue specialization at the metabolite level is perceived during the development of tomato fruit. J. Exp. Bot. 58, 4131–4146. doi: 10.1093/jxb/erm271 18065765

[B40] Mohanty B. Ong B. L. (2003). Contrasting effects of submergence in light and dark on pyruvate decarboxylase activity in roots of rice lines differing in submergence tolerance. Ann. Bot. 91, 291–300. doi: 10.1093/aob/mcf050 12509349 PMC4244983

[B41] Ngumbi E. Dady E. Calla B. (2022). Flooding and herbivory: the effect of concurrent stress factors on plant volatile emissions and gene expression in two heirloom tomato varieties. BMC Plant Biol. 22, 1–18. doi: 10.1186/s12870-022-03911-3 36396998 PMC9670554

[B42] Niu L. Jiang F. Yin J. Wang Y. Li Y. Yu X.. (2023). ROS-mediated waterlogging memory, induced by priming, mitigates photosynthesis inhibition in tomato under waterlogging stress. Front. Plant Sci. 14. doi: 10.3389/fpls.2023.1238108 PMC10493394 37701806

[B43] Oertel A. Matros A. Hartmann A. Arapitsas P. Dehmer K. J. Martens S.. (2017). Metabolite profiling of red and blue potatoes revealed cultivar and tissue specific patterns for anthocyanins and other polyphenols. Planta 246, 281–297. doi: 10.1007/s00425-017-2718-4 28664422

[B44] Pampana S. Masoni A. Arduini I. (2016). Response of cool-season grain legumes to waterlogging at flowering. Can. J. Plant Sci. 96, 597–603. doi: 10.1139/cjps-2015-0268

[B45] Pan J. Sharif R. Xu X. Chen X. (2021). Mechanisms of waterlogging tolerance in plants: Research progress and prospects. Front. Plant Sci. 11. doi: 10.3389/fpls.2020.627331 PMC7902513 33643336

[B46] Park S. U. Lee C. J. Kim S. E. Lim Y. H. Lee H. U. Nam S. S.. (2020). Selection of flooding stress tolerant sweetpotato cultivars based on biochemical and phenotypic characterization. Plant Physiol. Biochem. 155, 243–251. doi: 10.1016/j.plaphy.2020.07.039 32781274

[B47] Quinet M. Angosto T. Yuste-Lisbona F. J. Blanchard-Gros R. Bigot S. Martinez J. P.. (2019). Tomato fruit development and metabolism. Front. Plant Sci. 10. doi: 10.3389/fpls.2019.01554 PMC6895250 31850035

[B48] Rao R. Li Y. (2003). Management of flooding effects on growth of vegetable and selected field crops. HortTechnology 13, 610–616. doi: 10.21273/HORTTECH.13.4.0610

[B49] Rašeta S. Antić M. Todorović V. (2022b). Morphological diversity of tomato accessions from the Gene Bank of the Republic of Srpska. Agro-Knowledge Journal/Agroznanje 23, 1–11. doi: 10.7251/AGREN2201001R

[B50] Rašeta S. Kukavica B. Maksimović I. Mišić D. Antić M. (2022a). “Activities of Class III peroxidase and polyphenol oxidase in fruits of selected tomato genotypes from Gene Bank of Republic of Srpska,” in Book of Abstracts 4th International Conference on Plant Biology. Serbian Plant Physiology Society and the Institute for Bilogical Research Siniša Stanković - Natiolan Institute of the Republic of Serbia, University of Belgrade and the Faculty of Bilogy Univerity of Belgrade. 101.

[B51] Rašeta S. Stanivuković S. Kukavica B. Antić M. (2023). Biochemical characteristics of tomato landraces from Gene Bank of Republic of Srpska. J. Hygienic Eng. Design 42, 216–221.

[B52] Rasheed R. Iqbal M. Ashraf M. A. Hussain I. Shafiq F. Yousaf A.. (2018). Glycine betaine counteracts the inhibitory effects of waterlogging on growth, photosynthetic pigments, oxidative defence system, nutrient composition, and fruit quality in tomato. J. Hortic. Sci. Biotechnol. 93 (4), 385–391. doi: 10.1080/14620316.2017.1373037

[B53] Rice-Evans C. A. Miller N. J. Paganga G. (1996). Structure-antioxidant activity relationships of flavonoids and phenolic acids. Free Radical Biol. Med. 20, 933–956. doi: 10.1016/0891-5849(95)02227-9 8743980

[B54] Rosa-Martínez E. Bovy A. Plazas M. Tikunov Y. Prohens J. Pereira-Dias L. (2023). Genetics and breeding of phenolic content in tomato, eggplant and pepper fruits. Front. Plant Sci. 14. doi: 10.3389/fpls.2023.1135237 PMC10070870 37025131

[B55] Sasidharan R. Bailey-Serres J. Ashikari M. Atwell B. J. Colmer T. D. Fagerstedt K. (2017). Community recommendations on terminology and procedures used in flooding and low oxygen stress research. New Phytol. 214, 1403–1407. doi: 10.1111/nph.14519 28277605

[B56] Sauter M. (2013). Root responses to flooding. Curr. Opin. Plant Biol. 16, 282–286. doi: 10.1016/j.pbi.2013.03.013 23608517

[B57] Sergiev I. Alexieva V. Karanov E. (1997). Effect of spermine, atrazine and combination between them on some endogenous protective systems and stress markers in plants. Comptes Rendus l ‘Academie Bulg Des. Sci. 51, 121–124.

[B58] Siracusa L. Avola G. Patane C. Riggi E. Ruberto G. (2013). Re-evaluation of traditional Mediterranean foods. The local landraces of ‘Cipolla di Giarratana’ (*Allium cepa* L.) and long-storage tomato (*Lycopersicon esculentum* L.): quality traits and polyphenol content. J. Sci. Food Agric. 93, 3512–3519. doi: 10.1002/jsfa.6199 23633295

[B59] Šola I. Stić P. (2021). Effect of flooding and drought on the content of phenolics, sugars, photosynthetic pigments and vitamin C, and antioxidant potential of young Chinese cabbage. Eur. Food Res. Technol. 247, 1913–1920. doi: 10.1007/s00217-021-03759-1

[B60] Stadtherr L. Coumou D. Petoukhov V. Petri S. Rahmstorf S. (2016). Record Balkan floods of 2014 linked to planetary wave resonance. Sci. Adv. 2, e1501428. doi: 10.1126/sciadv.1501428 27152340 PMC4846427

[B61] Tamasi G. Pardini A. Bonechi C. Donati A. Pessina F. Marcolongo P.. (2019). Characterization of nutraceutical components in tomato pulp, skin and locular gel. Eur. Food Res. Technol. 245, 907–918. doi: 10.1007/s00217-019-03235-x

[B62] The Republic Hydrometeorological Institute (2022). Available online at: https://rhmzrs.com/ (Accessed February 14, 2024).

[B63] Tohge T. Fernie A. R. (2015). Metabolomics-inspired insight into developmental, environmental and genetic aspects of tomato fruit chemical composition and quality. Plant Cell Physiol. 56, 1681–1696. doi: 10.1093/pcp/pcv093 26228272

[B64] Trinh T. A. Feeny S. Posso A. (2021). “The impact of natural disasters and climate change on agriculture: Findings from Vietnam,” in Economic effects of natural disasters (Academic Press, Elsevier Inc. 2021). doi: 10.1016/B978-0-12-817465-4.00017-0

[B65] Tsao R. McCallum J. (2009). “Chemistry of flavonoids,” in Fruit and vegetable phytochemicals: chemistry, nutritional value and stability. Eds. de la Rosa L. A. Alvarez-Parrilla E. Gonzalez-Aguilar G. (Blackwell Publishing, Ames), 131–153, chapter 5.

[B66] Tutiempo Network S. L. (2022). Available online at: https://en.tutiempo.net/ (Accessed February 13, 2024).

[B67] Vallverdú-Queralt A. Jauregui O. Medina-Remón A. Andrés-Lacueva C. Lamuela-Raventós R. M. (2010). Improved characterization of tomato polyphenols using liquid chromatography/electrospray ionization linear ion trap quadrupole Orbitrap mass spectrometry and liquid chromatography/electrospray ionization tandem mass spectrometry. Rapid Commun. Mass Spectrometry 24, 2986–2992. doi: 10.1002/rcm.4731 20872631

[B68] Veljović Jovanović S. Kukavica B. Vidović M. Morina F. Menckhoff L. (2018). “Class III peroxidases: functions, localization and redox regulation of isoenzymes,” in Antioxidants and Antioxidant Enzymes in Higher Plants. Eds. Gupta D. Palma J. Corpas F. (Springer, Cham). doi: 10.1007/978-3-319-75088-0_13

[B69] Voesenek L. Colmer T. Pierik R. Millenaar F. Peeters A. (2006). How plants cope with complete submergence. New Phytol. 170, 213–226. doi: 10.1111/j.1469-8137.2006.01692.x 16608449

[B70] Walter S. Heuberger H. Schitzler W. H. (2004). “Sensibility of different vegetables to oxygen deficiency and aeration with H_2_O_2_ in the rhizosphere,” in VII International Symposium on Protected Cultivation in Mild Winter Climates: Production, Pest Management and Global Competition, Acta Horticulturae Vol. 659, 499–508.

[B71] Witt T. W. Flynn K. C. Villavicencio C. Northup B. K. (2022). Flood tolerance and flood loss predictions for tepary bean across the US Southern Great Plains. Agron. J. 114, 2169–2179. doi: 10.1002/agj2.21084

[B72] Wu S. B. Meyer R. S. Whitaker B. D. Litt A. Kennelly E. J. (2013). A new liquid chromatography–mass spectrometry-based strategy to integrate chemistry, morphology, and evolution of eggplant (*Solanum*) species. J. Chromatogr. 1314, 154–172. doi: 10.1016/j.chroma.2013.09.017 24055226

[B73] Yeboah M. A. Xuehao C. Guohua L. Minghong G. Chenwu X. (2008). Inheritance of waterlogging tolerance in cucumber (*Cucumis sativus* L.) Genetics of waterlogging in cucumber. Euphytica 162, 145–154. doi: 10.1007/s10681-007-9636-7

[B74] Yin J. Niu L. Li Y. Song X. Ottosen C. O. Wu Z.. (2023). The effects of waterlogging stress on plant morphology, leaf physiology and fruit yield in six tomato genotypes at anthesis stage. Vegetable Res. 3. doi: 10.48130/VR-2023-0031

[B75] Yiu J. C. Tseng M. J. Liu C. W. (2011). Exogenous catechin increases antioxidant enzyme activity and promotes flooding tolerance in tomato (*Solanum lycopersicum* L.). Plant Soil 344, 213–225. doi: 10.1007/s11104-011-0741-y

[B76] Yordanova R. Y. Christov K. N. Popova L. P. (2004). Antioxidative enzymes in barley plants subjected to soil flooding. Environ. Exp. Bot. 51, 93–101. doi: 10.1016/S0098-8472(03)00063-7

[B77] Zhang G. Tanakamaru K. Abe J. Morita S. (2007). Influence of waterlogging on some anti-oxidative enzymatic activities of two barley genotypes differing in anoxia tolerance. Acta Physiologiae Plantarum 29, 171–176. doi: 10.1007/s11738-006-0022-1

[B78] Zhou R. Jiang F. Yu X. Abdelhakim L. Li X. Rosenqvist E.. (2022). Dominant and Priming Role of Waterlogging in Tomato at e [CO_2_] by Multivariate Analysis. Int. J. Mol. Sci. 23, 12121. doi: 10.3390/ijms232012121 36292978 PMC9602540

[B79] Zhou R. Niu L. Yin J. Jiang F. Wang Y. Zhao T.. (2023). Differences in physiological responses of two tomato genotypes to combined waterlogging and cadmium stresses. Antioxidants 12, 1205. doi: 10.3390/antiox12061205 37371935 PMC10295130

