# Supplementary material for: Stress response in tomato as influenced by repeated waterlogging
Source: Front Plant Sci. 2024 Jul 23;15:1331281. doi: 10.3389/fpls.2024.1331281 (PMC11300220; doi:10.3389/fpls.2024.1331281)
Supplement: Supplementary file 1 [file Table_1.docx]

Supplementary Material

# Supplementary Tables

**SUPPLEMENTARY TABLE 1.** Temperatures (°C) registered over the 3-month period of the experiment logged every day on an outside meteorological station next to the greenhouse (Source: <https://rhmzrs.com/>).

| Date | May-22 | | | Jun-22 | | | Jul-22 | | |
| --- | --- | --- | --- | --- | --- | --- | --- | --- | --- |
|  | Tmin | Tmax | T mean | Tmin | Tmax | T mean | Tmin | Tmax | T mean |
| 1 | 8.3 | 22 | 15.5 | 12.1 | 28.7 | 22.4 | 20.8 | 38.2 | 30.3 |
| 2 | 13.5 | 19.4 | 14.0 | 15 | 30.6 | 24.2 | 20.7 | 31.8 | 26.2 |
| 3 | 5.7 | 21.3 | 13.3 | 16.9 | 30.4 | 25.5 | 19 | 34.2 | 27.5 |
| 4 | 5.1 | 24.8 | 16.4 | 20 | 33 | 26.0 | 20.8 | 36.1 | 26.4 |
| 5 | 7.7 | 24.8 | 17.6 | 18.6 | 33.5 | 26.9 | 22.1 | 31.8 | 25.1 |
| 6 | 10.7 | 20 | 15.2 | 18.6 | 25.2 | 21.5 | 17.1 | 31.4 | 23.9 |
| 7 | 12.2 | 18.2 | 14.4 | 14.8 | 29.1 | 23.3 | 14.9 | 30 | 22.9 |
| 8 | 11.5 | 23.1 | 16.8 | 16.6 | 24.8 | 19.5 | 16.6 | 22.2 | 18.9 |
| 9 | 12 | 25 | 18.4 | 16.5 | 24.1 | 18.5 | 17.3 | 26 | 20.0 |
| 10 | 10.3 | 26 | 17.3 | 16.7 | 23.1 | 19.5 | 13.9 | 26 | 20.2 |
| 11 | 9.8 | 28.2 | 19.2 | 18 | 25.2 | 20.1 | 14.2 | 25.5 | 19.6 |
| 12 | 10.2 | 31.1 | 22.7 | 14.2 | 27.9 | 21.7 | 11.3 | 28.2 | 21.4 |
| 13 | 13 | 30.4 | 23.0 | 13 | 30 | 23.2 | 13.4 | 29.8 | 22.5 |
| 14 | 17.2 | 27 | 20.1 | 17.9 | 26.3 | 20.5 | 14 | 35.6 | 26.1 |
| 15 | 9.8 | 28.3 | 20.2 | 11.8 | 29 | 21.7 | 15.2 | 34.1 | 26.4 |
| 16 | 10.5 | 31.2 | 23.4 | 12.9 | 31.8 | 24.0 | 16.4 | 32.1 | 25.2 |
| 17 | 15 | 27 | 18.8 | 18.2 | 29.7 | 23.6 | 18.6 | 29.7 | 23.3 |
| 18 | 13.8 | 23 | 16.2 | 14.8 | 28.1 | 21.4 | 13.8 | 30.8 | 22.4 |
| 19 | 5.7 | 26.8 | 17.0 | 12.9 | 31.1 | 23.1 | 13 | 32.3 | 23.6 |
| 20 | 7.2 | 31.9 | 21.1 | 14.1 | 35.3 | 26.2 | 14 | 35.1 | 25.4 |
| 21 | 13.3 | 31.8 | 23.7 | 18.8 | 34.9 | 28.0 | 15.4 | 37.2 | 26.9 |
| 22 | 14.4 | 28.2 | 22.6 | 19.9 | 30.3 | 25.5 | 17.2 | 38.9 | 29.4 |
| 23 | 17.2 | 27.9 | 21.9 | 21.6 | 29.8 | 25.5 | 18.9 | 40.8 | 31.0 |
| 24 | 14 | 32 | 24.6 | 18.5 | 33.6 | 26.4 | 21.2 | 31.9 | 25.7 |
| 25 | 14.1 | 31.8 | 24.3 | 18.8 | 31.5 | 24.1 | 18.9 | 36 | 28.0 |
| 26 | 17 | 27.3 | 21.7 | 16.9 | 33.9 | 26.2 | 17.8 | 36.3 | 24.7 |
| 27 | 15.9 | 31.9 | 24.6 | 17.2 | 36.2 | 28.1 | 18.6 | 31.8 | 23.5 |
| 28 | 13.9 | 23.2 | 15.3 | 19.8 | 36.3 | 28.8 | 18.9 | 31.5 | 24.7 |
| 29 | 12.8 | 18.9 | 14.5 | 19.8 | 36.8 | 28.3 | 16.8 | 35.9 | 27.5 |
| 30 | 11.9 | 16.6 | 14.4 | 19.9 | 36.2 | 29.4 | 19.9 | 31.1 | 24.2 |
| 31 | 13.8 | 24.1 | 18.5 |  |  |  | 15.6 | 30.7 | 23.3 |
| Mean | 11.9 | 25.9 | 18.9 | 16.8 | 30.5 | 24.1 | 17.0 | 32.4 | 24.7 |
| Min | 5.1 | 16.6 | 13.3 | 11.8 | 23.1 | 18.5 | 11.3 | 22.2 | 18.9 |
| Max | 17.2 | 32.0 | 24.6 | 21.6 | 36.8 | 29.4 | 22.1 | 40.8 | 31.0 |

**SUPPLEMENTARY TABLE 2.** Photoperiod (hours of daylight) in Banja Luka over the 3-month period of the experiment (Source: https://en.tutiempo.net/).

| Date | May-22 | Jun-22 | Jul-22 |
| --- | --- | --- | --- |
| 1 | 14:08 | 15:18 | 15:33 |
| 2 | 14:11 | 15:20 | 15:33 |
| 3 | 14:14 | 15:21 | 15:32 |
| 4 | 14:17 | 15:22 | 15:31 |
| 5 | 14:19 | 15:23 | 15:30 |
| 6 | 14:22 | 15:25 | 15:29 |
| 7 | 14:25 | 15:26 | 15:29 |
| 8 | 14:27 | 15:26 | 15:28 |
| 9 | 14:30 | 15:28 | 15:26 |
| 10 | 14:32 | 15:29 | 15:26 |
| 11 | 14:34 | 15:29 | 15:24 |
| 12 | 14:38 | 15:31 | 15:23 |
| 13 | 14:40 | 15:32 | 15:21 |
| 14 | 14:42 | 15:32 | 15:20 |
| 15 | 14:44 | 15:33 | 15:19 |
| 16 | 14:46 | 15:33 | 15:17 |
| 17 | 14:49 | 15:34 | 15:16 |
| 18 | 14:51 | 15:34 | 15:14 |
| 19 | 14:54 | 15:34 | 15:12 |
| 20 | 14:56 | 15:35 | 15:10 |
| 21 | 14:58 | 15:35 | 15:09 |
| 22 | 15:00 | 15:35 | 15:07 |
| 23 | 15:02 | 15:36 | 15:05 |
| 24 | 15:04 | 15:36 | 15:03 |
| 25 | 15:06 | 15:35 | 15:01 |
| 26 | 15:08 | 15:35 | 14:59 |
| 27 | 15:09 | 15:35 | 14:57 |
| 28 | 15:11 | 15:34 | 14:55 |
| 29 | 15:13 | 15:34 | 14:53 |
| 30 | 15:15 | 15:34 | 14:51 |
| 31 | 15:16 |  | 14:49 |

**SUPPLEMENTARY TABLE 3.** The content of major phenolic compounds in leaves of tomato varieties GB1126, GB1129 and NJ at seedling stage (57-60 day of growing) and at full flowering stage (75-78 day of growing). Data represent mean ± SEM and was analyzed by ANOVA followed by post hoc Tukey’s test.

|  | | Day of growing | | | | | |
| --- | --- | --- | --- | --- | --- | --- | --- |
| Phenol | Genotype | 57 day | 58 day | 60 day | 75 day | 76 day | 78 day |
| Caffeic acid | GB1126 | ND | ND | ND | 0,53 ± 0,10**e** | ND | ND |
|  | GB1129 | ND | ND | ND | ND | 0,25 ± 0,15**f** | ND |
|  | NJ | ND | ND | ND | ND | ND | ND |
| 3-0-Caffeoylquinic acid | GB1126 | 0,03 ± 0,00**e** | ND | 0,05 ± 0,00**g** | 1,43 ± 0,05**e** | 0,64 ± 0,02**f** | 0,30 ± 0,02**e** |
|  | GB1129 | 0,04 ± 0,01**e** | 0,02 ± 0,00**e** | 0,05 ± 0,01**g** | 0,67 ± 0,02**e** | 0,98 ± 0,01**f** | 0,22 ± 0,03**e** |
|  | NJ | 0,02 ± 0,00**e** | 0,05 ± 0,00**e** | 0,05 ± 0,01**g** | 1,43 ± 0,03**e** | 0,69 ± 0,02**f** | 0,73 ± 0,05**e** |
| 5-0-Caffeoylquinic acid | GB1126 | 3,88 ± 0,14**c** | 1,23 ± 0,05**d** | 2,49 ± 0,05**d** | 205,72 ± 7,67**a** | 70,14 ± 8,66**b** | 40,30 ± 5,21**c** |
|  | GB1129 | 4,16 ± 0,41**c** | 1,80 ± 0,16**d** | 2,05 ± 0,11**e** | 140,48 ± 1,67**b** | 101,57 ± 3,02**a** | 21,69 ± 2,86**d** |
|  | NJ | 1,60 ± 0,17**d** | 1,73 ± 0,05**d** | 1,59 ± 0,02**f** | 140,26 ± 1,85**b** | 71,29 ± 5,49**b** | 54,68 ± 4,61**b** |
| Quercetin 3-0-glucoside | GB1126 | 0,21 ± 0,03**e** | 0,22 ± 0,02**e** | 0,15 ± 0,02**g** | 1,10 ± 0,04**e** | 0,61 ± 0,06**f** | 0,89 ± 0,04**e** |
|  | GB1129 | 0,18 ± 0,03**e** | 0,21 ± 0,04**e** | 0,14 ± 0,04**g** | 1,18 ± 0,02**e** | 1,31 ± 0,01**f** | 1,37 ± 0,09**e** |
|  | NJ | 0,19 ± 0,02**e** | 0,15 ± 0,02**e** | 0,21 ± 0,05**g** | 1,20 ± 0,02**e** | 0,81 ± 0,02**f** | 0,72 ± 0,06**e** |
| Kaempferol 3-0-glucoside | GB1126 | 0,10 ± 0,04**e** | 0,13 ± 0,02**e** | 0,10 ± 0,03**g** | 0,31 ± 0,04**e** | 0,31 ± 0,02**f** | 0,45 ± 0,03**e** |
|  | GB1129 | 0,07 ± 0,05**e** | 0,09 ± 0,02**e** | 0,11 ± 0,05**g** | 0,51 ± 0,09**e** | 0,46 ± 0,04**f** | 0,72 ± 0,09**e** |
|  | NJ | 0,10 ± 0,02**e** | 0,10 ± 0,03**e** | 0,08 ± 0,02**g** | 0,33 ± 0,01**e** | 0,29 ± 0,03**f** | 0,22 ± 0,03**e** |
| Rutin | GB1126 | 9,77 ± 0,82**a** | 8,86 ± 0,50**a** | 7,13 ± 0,51**b** | 78,68 ± 2,67**c** | 34,72 ± 1,21**e** | 59,80 ± 2,74**b** |
|  | GB1129 | 9,27 ± 0,72**a** | 7,85 ± 0,45**b** | 5,44 ± 0,09**c** | 65,51 ± 2,72**d** | 62,00 ± 2,09**c** | 76,14 ± 5,29**a** |
|  | NJ | 6,14 ± 0,67**b** | 5,50 ± 0,38**c** | 9,92 ± 0,21**a** | 75,49 ± 2,28**c** | 48,40 ± 1,18**d** | 52,36 ± 3,57**b** |
| Naringin | GB1126 | ND | ND | ND | 0,58 ± 0,06**e** | 0,46 ± 0,11**f** | 0,44 ± 0,10**e** |
|  | GB1129 | ND | ND | ND | 0,30 ± 0,05**e** | 0,19 ± 0,05**f** | 0,20 ± 0,04**e** |
|  | NJ | ND | ND | ND | 0,27 ± 0,02**e** | 0,16 ± 0,07**f** | 0,38 ± 0,05**e** |

**SUPPLEMENTARY TABLE 4.** The content of major phenolic compounds in leaves of GB1126, GB1129, and NJ at the stage of fruit ripeness; Data represent mean ± SEM and was analyzed by ANOVA followed by post hoc Tukey’s test.

|  | Genotype | | |
| --- | --- | --- | --- |
|  | GB1126 | GB1129 | NJ |
| 3-O-Caffeoylquinic_acid | 35,93 ± 1,90 **d** | 5,50 ± 0,10 **c** | 29,10 ± 0,49 **c** |
| 5-O-Caffeoylquinic_acid | 852,04 ± 5,09 **b** | 647,60 ± 61,02 **b** | 1017,87 ± 40,36 **b** |
| Quercetin_3-O-glucoside | 58,70 ± 3,63 **c** | 68,14 ± 1,14 **c** | 87,44 ± 4,68 **c** |
| Kaempferol_3-O-glucoside | 6,21 ± 0,28 **e** | 9,10 ± 0,35 **c** | 7,18 ± 0,12 **c** |
| Rutin | 1226,15 ± 9,74 **a** | 1130,11 ± 14,65 **a** | 1405,54 ± 52,12 **a** |
| Naringenin | 0,32 ± 0,01 **e** | 0,39 ± 0,04 **c** | 0,40 ± 0,01 **c** |
| Naringin | 7,00 ± 0,47 **e** | 1,76 ± 0,17 **c** | 6,28 ± 0,60 **c** |
| Eriodictyol | 0,51 ± 0,04 **e** | 0,81 ± 0,05 **c** | 0,90 ± 0,05 **c** |

**SUPPLEMENTARY TABLE 5.** The content of major phenolic compounds in fruits of GB1126, GB1129, and NJ at the stage of fruit ripeness; Data represent mean ± SEM and was analyzed by ANOVA followed by post hoc Tukey’s test.

|  | Genotype | | |
| --- | --- | --- | --- |
|  | GB1126 | GB1129 | NJ |
| Caffeic_acid | 3,70 ± 0,29 e | 5,14 ± 0,41 c | 3,44 ± 0,06 d |
| 3-O-Caffeoylquinic_acid | 39,34 ± 1,27 d | 46,31 ± 1,52 c | 16,80 ± 1,38 d |
| 5-O-Caffeoylquinic_acid | 260,49 ± 26,72 a | 223,29 ± 9,57 a | 212,48 ± 4,85 b |
| Luteolin | 6,48 ± 0,11 e | 4,88 ± 1,20 c | 6,45 ± 0,22 d |
| Apigenin | 184,88 ± 7,22 c | 170,79 ± 28,53 b | 162,87 ± 14,10 c |
| Quercetin_3-O-glucoside | 1,48 ± 0,10 e | 1,45 ± 0,13 c | 2,52 ± 0,61 d |
| Rutin | 222,59 ± 8,24 b | 261,83 ± 21,86 a | 259,12 ± 3,52 a |
| Naringenin | 183,31 ± 8,65 c | 168,13 ± 26,70 b | 164,87 ± 10,82 c |
| Naringin | 0,71 ± 0,06 e | 0,58 ± 0,04 c | 0,70 ± 0,10 d |
| Eriodictyol | 10,98 ± 0,02 e | 7,63 ± 1,38 c | 10,28 ± 0,92 d |

**SUPPLEMENTARY TABLE 6.** Genotype-dependent phenolic profiles in leaves of GB1126 at seedling and full-flowering stage as influenced by waterlogging. Data represent mean ± SEM and was analyzed by ANOVA followed by post hoc Tukey’s test.

| Phenol | Growth regime | Day of growing | | | | | |
| --- | --- | --- | --- | --- | --- | --- | --- |
|  |  | 57 day | 58 day | 60 day | 75 day | 76 day | 78 day |
| Caffeic acid | C | ND | ND | ND | 0,53 ± 0,10**g** | ND | ND |
|  | T2 | ND | ND | ND | 0,31 ± 0,01**g** | ND | ND |
|  | T1 | ND | ND | ND | 0,33 ± 0,02**g** | ND | ND |
| 3-0-Caffeoylquinic acid | C | 0,03 ± 0,00**c** | ND | 0,05 ± 0,00**e** | 1,43 ± 0,05**g** | 0,64 ± 0,02**d** | 0,30 ± 0,02**f** |
|  | T2 | 0,03 ± 0,00**c** | 0,02 ± 0,00**d** | ND | 1,55 ± 0,05**g** | 0,40 ± 0,03**d** | 0,24 ± 0,02**f** |
|  | T1 | 0,03 ± 0,00**c** | ND | 0,05 ± 0,00**e** | 1,73 ± 0,04**g** | 0,24 ± 0,01**d** | 0,31 ± 0,01**f** |
| 5-0-Caffeoylquinic acid | C | 3,88 ± 0,14**b** | 1,23 ± 0,05**c** | 2,49 ± 0,05**c** | 205,72 ± 7,67**a** | 70,14 ± 8,66**a** | 40,30 ± 5,21**c** |
|  | T2 | 3,88 ± 0,14**b** | 1,57 ± 0,08**c** | 1,60 ± 0,06**d** | 142,63 ± 3,06**c** | 33,80 ± 7,00**b** | 27,96 ± 3,42**e** |
|  | T1 | 3,88 ± 0,14**b** | 1,23 ± 0,05**c** | 2,49 ± 0,05**c** | 157,35 ± 5,24**b** | 34,58 ± 3,17**b** | 33,82 ± 4,38**d** |
| Quercetin 3-0-glucoside | C | 0,21 ± 0,03**c** | 0,22 ± 0,02**d** | 0,15 ± 0,02**e** | 1,10 ± 0,04**g** | 0,61 ± 0,06**d** | 0,89 ± 0,04**f** |
|  | T2 | 0,21 ± 0,03**c** | 0,28 ± 0,01**d** | 0,20 ± 0,02**e** | 0,72 ± 0,03**g** | 0,28 ± 0,03**d** | 0,73 ± 0,03**f** |
|  | T1 | 0,21 ± 0,03**c** | 0,22 ± 0,02**d** | 0,15 ± 0,02**e** | 0,62 ± 0,01**g** | 0,38 ± 0,02**d** | 0,63 ± 0,01**f** |
| Kaempferol 3-0-glucoside | C | 0,10 ± 0,04**c** | 0,13 ± 0,02**d** | 0,10 ± 0,03**e** | 0,31 ± 0,04**g** | 0,31 ± 0,02**d** | 0,45 ± 0,03**f** |
|  | T2 | 0,10 ± 0,04**c** | 0,12 ± 0,02**d** | 0,14 ± 0,02**e** | 0,30 ± 0,05**g** | 0,17 ± 0,02**d** | 0,36 ± 0,07**f** |
|  | T1 | 0,10 ± 0,04**c** | 0,13 ± 0,02**d** | 0,10 ± 0,03**e** | 0,29 ± 0,03**g** | 0,17 ± 0,05**d** | 0,33 ± 0,09**f** |
| Rutin | C | 9,77 ± 0,82**a** | 8,86 ± 0,50**b** | 7,13 ± 0,51**b** | 78,68 ± 2,67**d** | 34,72 ± 1,21**b** | 59,80 ± 2,74**a** |
|  | T2 | 9,77 ± 0,82**a** | 19,12 ± 0,69**a** | 8,58 ± 0,44**a** | 47,53 ± 1,48**e** | 16,50 ± 0,77**c** | 48,43 ± 1,30**b** |
|  | T1 | 9,77 ± 0,82**a** | 8,86 ± 0,50**b** | 7,13 ± 0,51**b** | 38,74 ± 1,10**f** | 15,49 ± 0,65**c** | 29,14 ± 0,99**e** |
| Naringin | C | ND | ND | ND | 0,58 ± 0,06**g** | 0,46 ± 0,11**d** | 0,44 ± 0,10**f** |
|  | T2 | ND | ND | ND | 0,72 ± 0,06**g** | 0,55 ± 0,08**d** | 0,61 ± 0,07**f** |
|  | T1 | ND | ND | ND | 0,82 ± 0,04**g** | 0,50 ± 0,02**d** | 1,00 ± 0,10**f** |

**SUPPLEMENTARY TABLE 7.** Genotype-dependent phenolic profiles in leaves of GB1129 at seedling and full-flowering stage as influenced by waterlogging. Data represent mean ± SEM and was analyzed by ANOVA followed by post hoc Tukey’s test.

| Phenol | Growth regime | Day of growing | | | | | |
| --- | --- | --- | --- | --- | --- | --- | --- |
|  |  | 57 day | 58 day | 60 day | 75 day | 76 day | 78 day |
| Caffeic acid | C | ND | ND | ND | ND | 0,25 ± 0,15**e** | ND |
|  | T2 | ND | ND | ND | ND | ND | 0,22 ± 0,11**e** |
|  | T1 | ND | ND | ND | 0,13 ± 0,01**e** | 0,19 ± 0,07**e** | ND |
| 3-0-Caffeoylquinic acid | C | 0,04 ± 0,01**c** | 0,02 ± 0,00**d** | 0,05 ± 0,01**d** | 0,67 ± 0,02**e** | 0,98 ± 0,01**e** | 0,22 ± 0,03**e** |
|  | T2 | 0,04 ± 0,01**c** | 0,04 ± 0,00**d** | 0,02 ± 0,00**d** | 0,56 ± 0,02**e** | 0,24 ± 0,02**e** | 1,00 ± 0,03**e** |
|  | T1 | 0,04 ± 0,01**c** | 0,02 ± 0,00**d** | 0,05 ± 0,01**d** | 0,57 ± 0,02**e** | 0,17 ± 0,01**e** | ND |
| 5-0-Caffeoylquinic acid | C | 4,16 ± 0,41**b** | 1,80 ± 0,16**c** | 2,05 ± 0,11**c** | 140,48 ± 1,67**a** | 101,57 ± 3,02**a** | 21,69 ± 2,86**c** |
|  | T2 | 4,16 ± 0,41**b** | 1,46 ± 0,04**c** | 1,74 ± 0,06**c** | 80,38 ± 3,23**c** | 17,30 ± 6,65**d** | 115,00 ± 9,27**a** |
|  | T1 | 4,16 ± 0,41**b** | 1,80 ± 0,16**c** | 2,05 ± 0,11**c** | 92,91 ± 1,11**b** | 16,22 ± 1,53**d** | 9,88 ± 1,14**d** |
| Quercetin 3-0-glucoside | C | 0,18 ± 0,03**c** | 0,21 ± 0,04**d** | 0,14 ± 0,04**d** | 1,18 ± 0,02**e** | 1,31 ± 0,01**e** | 1,37 ± 0,09**e** |
|  | T2 | 0,18 ± 0,03**c** | 0,25 ± 0,03**d** | 0,27 ± 0,04**d** | 1,97 ± 0,07**e** | 1,22 ± 0,08**e** | 1,59 ± 0,07**e** |
|  | T1 | 0,18 ± 0,03**c** | 0,21 ± 0,04**d** | 0,14 ± 0,04**d** | 1,01 ± 0,03**e** | 1,02 ± 0,03**e** | 1,44 ± 0,09**e** |
| Kaempferol 3-0-glucoside | C | 0,07 ± 0,05**c** | 0,09 ± 0,02**d** | 0,11 ± 0,05**d** | 0,51 ± 0,09**e** | 0,46 ± 0,04**e** | 0,72 ± 0,09**e** |
|  | T2 | 0,07 ± 0,05**c** | 0,09 ± 0,04**d** | 0,13 ± 0,04**d** | 0,72 ± 0,02**e** | 0,77 ± 0,09**e** | 0,47 ± 0,06**e** |
|  | T1 | 0,07 ± 0,05**c** | 0,09 ± 0,02**d** | 0,11 ± 0,05**d** | 0,42 ± 0,02**e** | 0,62 ± 0,02**e** | 0,59 ± 0,02**e** |
| Rutin | C | 9,27 ± 0,72**a** | 7,85 ± 0,45**b** | 5,44 ± 0,09**b** | 65,51 ± 2,72**d** | 62,00 ± 2,09**b** | 76,14 ± 5,29**b** |
|  | T2 | 9,27 ± 0,72**a** | 11,98 ± 0,47**a** | 13,14 ± 0,60**a** | 93,76 ± 2,84**b** | 59,94 ± 2,35**b** | 83,47 ± 1,96**b** |
|  | T1 | 9,27 ± 0,72**a** | 7,85 ± 0,45**b** | 5,44 ± 0,09**b** | 67,42 ± 3,08**d** | 54,77 ± 2,53**c** | 79,73 ± 3,94**b** |
| Naringin | C | ND | ND | ND | 0,30 ± 0,05**e** | 0,19 ± 0,05**e** | 0,20 ± 0,04**e** |
|  | T2 | ND | ND | ND | 0,34 ± 0,08**e** | 0,17 ± 0,05**e** | 0,26 ± 0,06**e** |
|  | T1 | ND | ND | ND | 0,27 ± 0,09**e** | 0,18 ± 0,04**e** | 0,21 ± 0,02**e** |

**SUPPLEMENTARY TABLE 8.** Genotype-dependent phenolic profiles in leaves of NJ at seedling and full-flowering stage as influenced by waterlogging. Data represent mean ± SEM and was analyzed by ANOVA followed by post hoc Tukey’s test.

| Phenol | Growth regime | Day of growing | | | | | |
| --- | --- | --- | --- | --- | --- | --- | --- |
|  |  | 57 day | 58 day | 60 day | 75 day | 76 day | 78 day |
| Caffeic acid | C | ND | ND | ND | ND | ND | ND |
|  | T2 | ND | ND | ND | 0,16 ± 0,01**f** | ND | 0,15 ± 0,15**f** |
|  | T1 | ND | ND | ND | ND | 0,58 ± 0,11**e** | ND |
| 3-0-Caffeoylquinic acid | C | 0,02 ± 0,00**c** | 0,05 ± 0,00**d** | 0,05 ± 0,01**d** | 1,43 ± 0,03**f** | 0,69 ± 0,02**e** | 0,73 ± 0,05**f** |
|  | T2 | 0,02 ± 0,00**c** | 0,03 ± 0,00**d** | 0,05 ± 0,00**d** | 1,12 ± 0,03**f** | 0,95 ± 0,00**e** | 0,21 ± 0,01**f** |
|  | T1 | 0,02 ± 0,00**c** | 0,05 ± 0,00**d** | 0,05 ± 0,01**d** | 0,50 ± 0,01**f** | 0,70 ± 0,02**e** | 0,36 ± 0,01**f** |
| 5-0-Caffeoylquinic acid | C | 1,60 ± 0,17**b** | 1,73 ± 0,05**c** | 1,59 ± 0,02**c** | 140,26 ± 1,85**a** | 71,29 ± 5,49**b** | 54,68 ± 4,61**b** |
|  | T2 | 1,60 ± 0,17**b** | 1,74 ± 0,05**c** | 1,84 ± 0,06**c** | 98,21 ± 4,93**b** | 63,11 ± 3,31**c** | 10,74 ± 2,10**e** |
|  | T1 | 1,60 ± 0,17**b** | 1,73 ± 0,05**c** | 1,59 ± 0,02**c** | 47,84 ± 3,12**e** | 45,60 ± 1,25**d** | 28,45 ± 2,16**d** |
| Quercetin 3-0-glucoside | C | 0,19 ± 0,02**c** | 0,15 ± 0,02**d** | 0,21 ± 0,05**d** | 1,20 ± 0,02**f** | 0,81 ± 0,02**e** | 0,72 ± 0,06**f** |
|  | T2 | 0,19 ± 0,02**c** | 0,19 ± 0,04**d** | 0,25 ± 0,02**d** | 1,29 ± 0,02**f** | 1,05 ± 0,04**e** | 0,74 ± 0,03**f** |
|  | T1 | 0,19 ± 0,02**c** | 0,15 ± 0,02**d** | 0,21 ± 0,05**d** | 0,98 ± 0,03**f** | 1,67 ± 0,07**e** | 1,12 ± 0,04**f** |
| Kaempferol 3-0-glucoside | C | 0,10 ± 0,02**c** | 0,10 ± 0,03**d** | 0,08 ± 0,02**d** | 0,33 ± 0,01**f** | 0,29 ± 0,03**e** | 0,22 ± 0,03**f** |
|  | T2 | 0,10 ± 0,02**c** | 0,09 ± 0,04**d** | 0,07 ± 0,02**d** | 0,38 ± 0,03**f** | 0,48 ± 0,10**e** | 0,54 ± 0,02**f** |
|  | T1 | 0,10 ± 0,02**c** | 0,10 ± 0,03**d** | 0,08 ± 0,02**d** | 0,47 ± 0,07**f** | 0,76 ± 0,04**e** | 0,55 ± 0,08**f** |
| Rutin | C | 6,14 ± 0,67**a** | 5,50 ± 0,38**b** | 9,92 ± 0,21**b** | 75,49 ± 2,28**c** | 48,40 ± 1,18**d** | 52,36 ± 3,57**b** |
|  | T2 | 6,14 ± 0,67**a** | 9,77 ± 0,15**a** | 13,48 ± 1,17**a** | 76,82 ± 2,34**c** | 51,33 ± 0,37**d** | 38,47 ± 2,25**c** |
|  | T1 | 6,14 ± 0,67**a** | 5,50 ± 0,38**b** | 9,92 ± 0,21**b** | 61,54 ± 1,21**d** | 79,65 ± 2,03**a** | 59,25 ± 1,87**a** |
| Naringin | C | ND | ND | ND | 0,27 ± 0,02**f** | 0,16 ± 0,07**e** | 0,38 ± 0,05**f** |
|  | T2 | ND | ND | ND | 0,37 ± 0,02**f** | 0,31 ± 0,02**e** | 0,31 ± 0,05**f** |
|  | T1 | ND | ND | ND | 0,47 ± 0,03**f** | 0,10 ± 0,02**e** | 0,66 ± 0,12**f** |
